# Structures suggest a mechanism for energy coupling by a family of organic anion transporters

**DOI:** 10.1371/journal.pbio.3000260

**Published:** 2019-05-13

**Authors:** Jonathan B. Leano, Samir Batarni, Jacob Eriksen, Narinobu Juge, John E. Pak, Tomomi Kimura-Someya, Yaneth Robles-Colmenares, Yoshinori Moriyama, Robert M. Stroud, Robert H. Edwards

**Affiliations:** 1 Department of Biochemistry & Biophysics, University of California San Francisco School of Medicine, San Francisco, California, United States of America; 2 Departments of Neurology and Physiology, University of California San Francisco School of Medicine, San Francisco, California, United States of America; 3 Department of Membrane Biochemistry, Advanced Science Research Center, Okayama University, Okayama, Japan; University of Zurich, SWITZERLAND

## Abstract

Members of the solute carrier 17 (SLC17) family use divergent mechanisms to concentrate organic anions. Membrane potential drives uptake of the principal excitatory neurotransmitter glutamate into synaptic vesicles, whereas closely related proteins use proton cotransport to drive efflux from the lysosome. To delineate the divergent features of ionic coupling by the SLC17 family, we determined the structure of *Escherichia coli* D-galactonate/H^+^ symporter D-galactonate transporter (DgoT) in 2 states: one open to the cytoplasmic side and the other open to the periplasmic side with substrate bound. The structures suggest a mechanism that couples H^+^ flux to substrate recognition. A transition in the role of H^+^ from flux coupling to allostery may confer regulation by trafficking to and from the plasma membrane.

## Abstract 2

The first structures of a family of organic anion transporters reveal an interaction with protons that is conserved from bacterial transporters to the mammalian proteins that transport glutamate into synaptic vesicles.

## Introduction

Secondary active transporters couple the movement of ions down their electrochemical gradient to the concentration of substrate. Differences in the mechanism of energy coupling enable them to meet diverse physiological requirements. In general, transporters related in sequence exhibit similar ionic coupling. However, a highly conserved transporter family contains members that use distinct mechanisms of energy coupling to transport substrate in opposite directions, suggesting divergence from a common underlying mechanism.

The concentration of organic anions has a crucial role in diverse biological processes, from nutrient uptake by cells to the packaging of neurotransmitter into secretory vesicles for subsequent release by exocytosis. Members of the solute carrier 17 (SLC17) family catalyze the coupled transport of anions driven by a proton electrochemical gradient Δμ_H+_ (= Δψ − 2.3× (RT/F) × ΔpH, where R is the gas constant, T is temperature, and F is Faraday’s constant), with contributions from both electrical potential Δψ and proton gradient ΔpH (pH_o_/pH_i_). To take advantage of environmental conditions, members of the SLC17 family have tuned their response to the available driving force, illustrating an unusual form of adaptive divergence.

**Fig 1:**
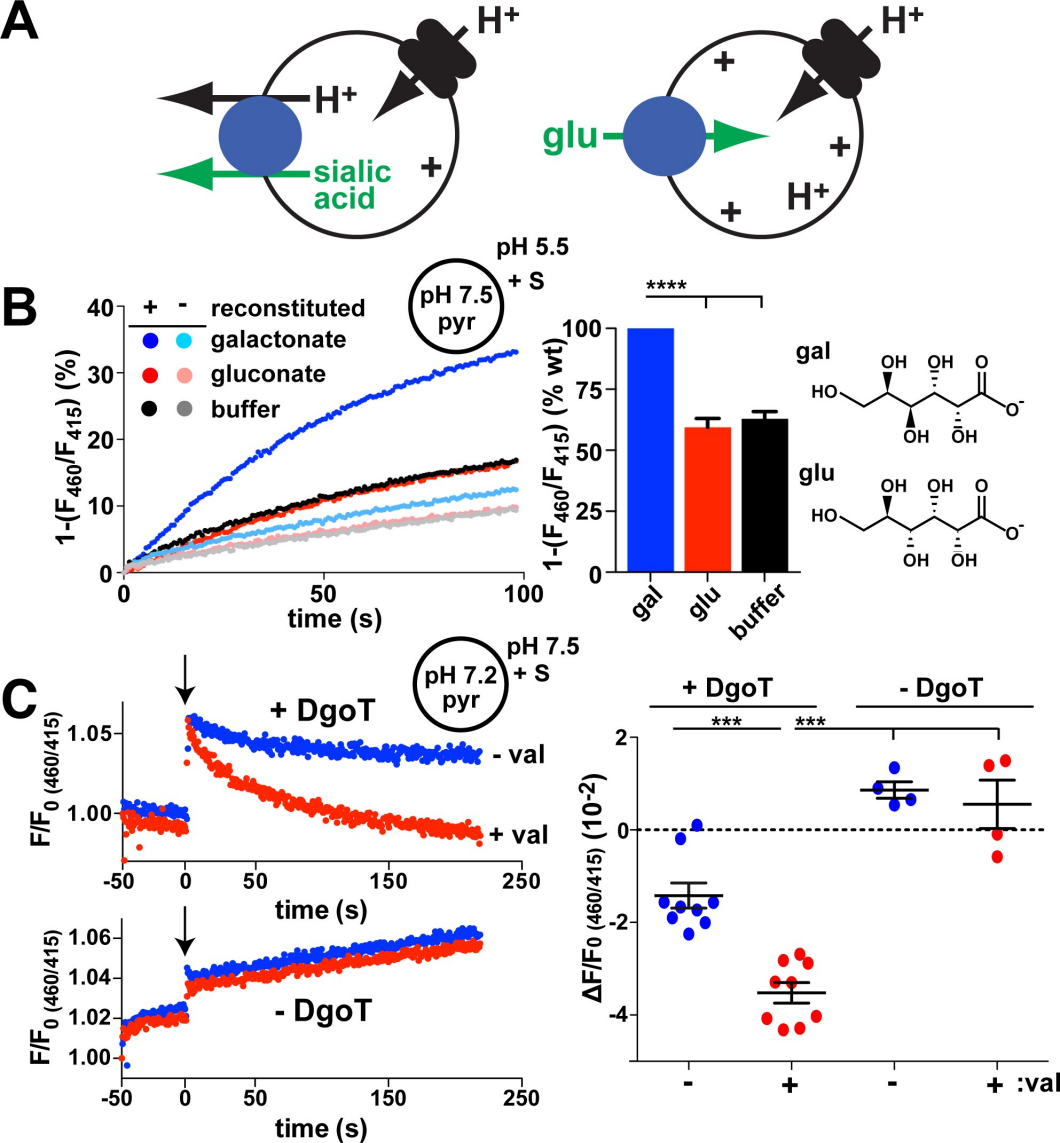
Coupled D-gal:H^+^ cotransport by *E*. *coli* DgoT. (A) Diagram illustrating transport by sialin (left) and a VGLUT (right). A vacuolar-type H^+^ pump provides the H^+^ electrochemical driving force for both activities, but sialin uses the pH gradient to drive electroneutral cotransport of sialic acid with H^+^ out of the lysosome whereas the VGLUTs rely on membrane potential to drive glutamate uptake by synaptic vesicles against a pH gradient. (B, C) Liposomes containing pyr at pH 7.5 were reconstituted with (+) or without (−) *E*. *coli* DgoT and added to reaction buffer at pH 5.5. gal, glu (both 10 mM), or buffer alone were added at *t* = 0. The fluorescence emission at 510 nm with excitation at 460 nm was normalized to excitation at 415 nm (the isosbestic point for pyr versus pH). (B) The representative change in fluorescence ratio shows that D-gal but not glu or buffer control reduce the lumenal pH of proteoliposomes reconstituted with DgoT but not empty liposomes. Bar graph (right) shows the average rate of fluorescence change for glu and buffer relative to gal (*n* = 6). ****p < 0.0001. (C) Traces (left) show representative fluorescence ratios of proteoliposomes with (above) and without DgoT (below), both formed at pH 7.2, before and after the addition of 10 mM D-gal (at arrow) in external solution at pH 7.5, with or without 0.2 μM val. Both internal and external solutions contained 5 mM K^+^. Scatterplot (right) shows the difference of fluorescence decay between the addition of gal (*t* = 0) and the end of recording (*t* ≈ 220). ***p < 0.001 by one-way ANOVA with Bonferroni correction. *n* = 9 for proteoliposomes with DgoT; *n* = 4 for liposomes (see S1 Fig). The numerical data underlying this figure are included in S1 Data. DgoT, D-galactonate transporter; gal, galactonate; glu, gluconate; pyr, pyranine; val, valinomycin; VGLUT, vesicular glutamate transporter; WT, wild type.

SLC17 protein sialin uses H^+^ symport [1, 2] to drive the anion sialic acid out of lysosomes (Fig 1A). The electroneutrality that derives from coupling to H^+^ enables the efflux of negatively charged sialic acid despite a lumen-positive Δψ. In contrast, vesicular glutamate transporters (VGLUTs), also of the SLC17 family, rely on membrane potential Δψ (inside positive) to accumulate the principal excitatory transmitter glutamate inside synaptic vesicles (Fig 1A) [3–6]. In this case, glutamate uptake occurs against the outwardly directed H^+^ gradient. The VGLUTs thus exhibit a dependence on components of Δμ_H+_ different from that of other SLC17 family members such as sialin. Sialin has itself been reported to confer vesicular transport of aspartate and glutamate [7, 8]. Although the physiological significance remains uncertain [9, 10], this finding would suggest that a single protein can catalyze both activities. The VGLUTs also differ from other vesicular neurotransmitter transporters (not of the SLC17 family) that act as H^+^ exchangers [11]. They nonetheless retain a pH dependence that limits their activity to uptake by acidic compartments such as synaptic vesicles and prevents nonvesicular glutamate efflux across the plasma membrane, where the pH is neutral [12].

How do different components of Δμ_H+_ drive transport in different directions through closely related proteins? Mammalian transport proteins of the SLC17 family show up to 27% sequence identity to bacterial relatives. Although of undefined function, many of the bacterial SLC17 genes occur within operons dedicated to the metabolism of acidic sugars such as galactonic, glucaric, and galacturonic acids [13]. To understand how the SLC17 family has adapted to divergent roles in metabolism and neurotransmitter transport, we determined the crystal structures of a D-galactonate transporter in 2 conformers.

## Results

### DgoT is a proton symporter highly selective for D-galactonate

The D-galactonate transporter (DgoT) gene lies within an operon devoted to the metabolism of D-galactonate, suggesting a role in transport of this acidic sugar [14]. Because the ionic coupling was not known, we reconstituted purified, recombinant DgoT into liposomes containing the pH-sensitive fluorophore 8-hydroxypyrene-trisulfonate (pyranine; S1 Fig). With an inwardly directed H^+^ gradient, addition of D-galactonate outside quenches the fluorescence of lumenal pyranine, indicating galactonate/H^+^ cotransport (Fig 1B). Without DgoT, without D-galactonate, or with the galactonate epimer gluconate, there is no effect on the pyranine fluorescence of proteoliposomes, suggesting high selectivity for galactonate. Because entry of the anion galactonate might alone create the inwardly negative Δψ that drives H^+^ influx, we tested the coupling between galactonate and H^+^ by adding galactonate to proteoliposomes with an outwardly directed H^+^ gradient (Fig 1C). Under these conditions, galactonate still quenches the pyranine fluorescence of proteoliposomes but not control liposomes, indicating that translocation of H^+^ and galactonate are coupled by DgoT, not by changes in Δψ. Further excluding a role for Δψ in the acidification by galactonate, dissipation of Δψ by the K^+^ ionophore valinomycin increases fluorescence quenching by galactonate (Figs 1C and S1). Indeed, the stimulation by valinomycin shows that Δψ opposes galactonate/H^+^ influx, suggesting that DgoT moves net charge, presumably positive. The transport activity demonstrates that purified DgoT is functional, enabling its use for structural studies. In addition, the H^+^ cotransport mechanism indicates similarity to the lysosomal sialic acid/H^+^ cotransporter sialin. Unlike electroneutral sialin, however, the effect of membrane potential on DgoT activity suggests that galactonate flux is coupled to the movement of more than one H^+^.

### Open inward structure of wild-type DgoT

To determine the structure of DgoT, the wild type (WT) protein was crystallized at pH 5.35, in an inward-open conformation and crystal space group P2_1_. Nonsubstrate gluconate improved the diffraction, and it as well as β-nonylglucoside (β-NG) were identified nonspecifically within the aqueous cavity of the structure (S2 Fig). The structure was determined to a resolution of 2.9 × 3.8 × 3.8 Å by molecular replacement (MR) trials based on all available major facilitator superfamily (MFS) transporters in turn because the MFS typically each crystallize in different parts of the transport cycle. The correct MR solution derived from a polyalanine model of (inward facing) Glycerol-3-phosphate transporter (GlpT; 1PW4) [16] and was subsequently built and refined to an R-factor/Rfree of 24.9%/29.7% (S1 Table). The asymmetric unit contains 2 almost identical, antiparallel monomers of DgoT, with 4 molecules of β-nonylglucoside (β-NG) between the 2 molecules of DgoT and a root-mean-square deviation (rmsd) of 0.57 Å between their congruent 392 Cα atoms.

**Fig 2:**
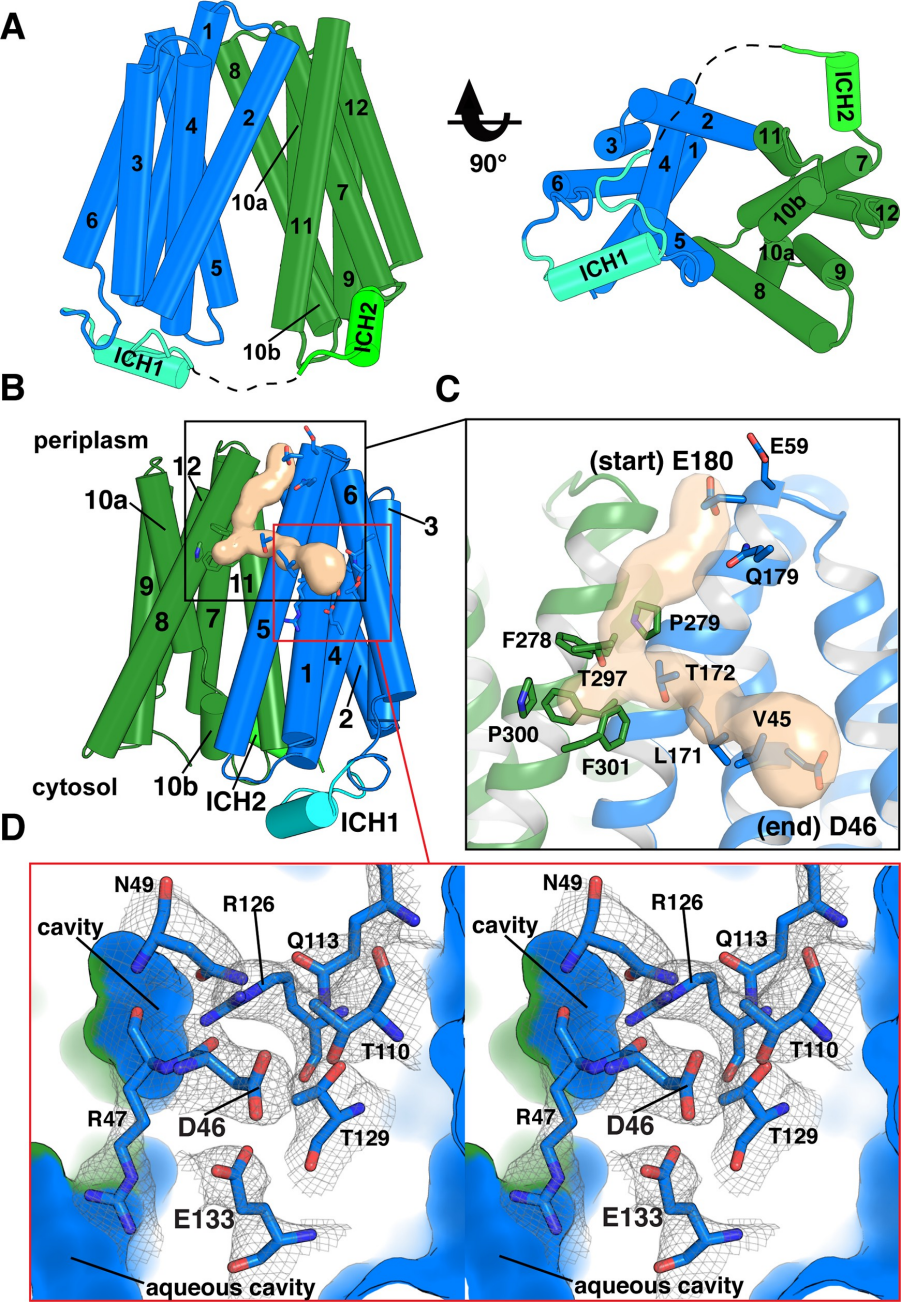
The inward-open structure of DgoT shows a proton translocation pathway connected to the periplasm. (A) Cylinder representation of inward-facing DgoT viewed parallel to the membrane plane and from the cytoplasm. Helices are numbered from the N to C terminus. Dotted line represents unstructured regions. (B) Overall view of inward-facing DgoT in cylinder representation shows the putative water tunnel (tan surface) with the surrounding residues shown as sticks. (C) A close-up view of the tunnel highlights the entrance and exit points. (D) Putative sites of protonation (Asp46 and Glu133) lie in a membrane-embedded pocket of polar residues buried within the NTD (crossed-eye stereo). The polar and charged residues composing the pocket are shown as sticks. Electron density is from a composite, simulated annealing “omit map” to eliminate model bias (1σ). Outer surface and cavities formed from interdomain contacts are shown in blue (NTD) and green (CTD). Asp46, aspartate-46; CTD, C-terminal domain; DgoT, D-galactonate transporter; Glu133, glutamate-133; ICH, intracellular helix; NTD, N-terminal domain.

The structure contains residues 27 through 443, lacking a 6–amino acid unstructured cytoplasmic loop (residues 235–242) between transmembrane (TM) domains 6 and 7 (Fig 2A). DgoT is composed of 12 TM helices and belongs to the MFS fold [17]. Two 6-helix bundles (N- and C-terminal domains) are related by a pseudo 2-fold symmetry axis perpendicular to the membrane plane. The DgoT structure has 2 intracellular helices (ICH1 and ICH2) between N- and C-terminal domains, similar to sugar transporters of the MFS [18–20]. The periplasmic ends of TM1, 2, and 5 from the N terminus and TM7, 8, and 11 from the C terminus form contacts that seal the aqueous cavity from the periplasmic side.

### Proton flux to an N-terminal polar pocket

Although the main cavity of the inwardly oriented structure is sealed to the outside, the N-terminal lobe of DgoT contains a tunnel that extends from the periplasmic surface near glutamate-180 (Glu180; TM5) to a membrane-embedded site at aspartate-46 (Asp46; TM1; Figs 2B, 2C and S4). The average diameter of the tunnel near its entrance (3.6 Å) and exit (5.0 Å) suggests accessibility to a line of water molecules. Polar residues lining the entrance (Glu59, glutamine-179 [Gln179], Glu180), interior (threonine-172 [Thr172], Thr297), and exit (Asp46) have the potential to interact with H_3_O^+^ and could therefore facilitate H^+^ transport. On the other hand, residues phenylalanine-278 (Phe278), proline-279 (Pro279), and Thr297 reduce the tunnel diameter to 2.4 Å, about the diameter of a water molecule (2.75 Å; Fig 2C), suggesting a dynamic mechanism to control access of H_3_O^+^ from the periplasm. A positively charged region near Pro279 will also raise the energy barrier for H^+^ movement toward the site of potential protonation at Asp46 (S4 Fig). With its side chain carboxyl sequestered, Asp46 is predicted to have a pKa of approximately 6.0 (H++ server) [21], and this could be much higher due to screening by the lipid bilayer. Thus, Asp46 is most probably protonated (uncharged) in this structure (Fig 2).

At the end of the tunnel, a pocket of conserved polar residues buried within the membrane-spanning region of the N-terminal domain lies adjacent to Asp46 (Fig 2C and 2D). In this pocket, residues conserved between sialin and the VGLUTs include arginine-47 (Arg47; TM1), Arg126, and Glu133 (TM4). However, Asp46 itself is not conserved in metazoan SLC17 proteins, suggesting it may contribute to the difference in function between family members. The buried Glu133 residue has a predicted pKa of approximately 8.1 (H++ server) [21], suggesting that it is protonated. Although Arg47 is directly adjacent, the structure thus indicates that the 2 residues do not form a charge pair in this inward-facing state. Multiple TM helices contribute less well conserved residues to this unusual charged pocket buried in the *trans*-bilayer region, including asparagine-49 (Asn49; TM1), Thr110, Gln113 (TM3), and Thr129 (TM4; Fig 2D).

**Fig 3:**
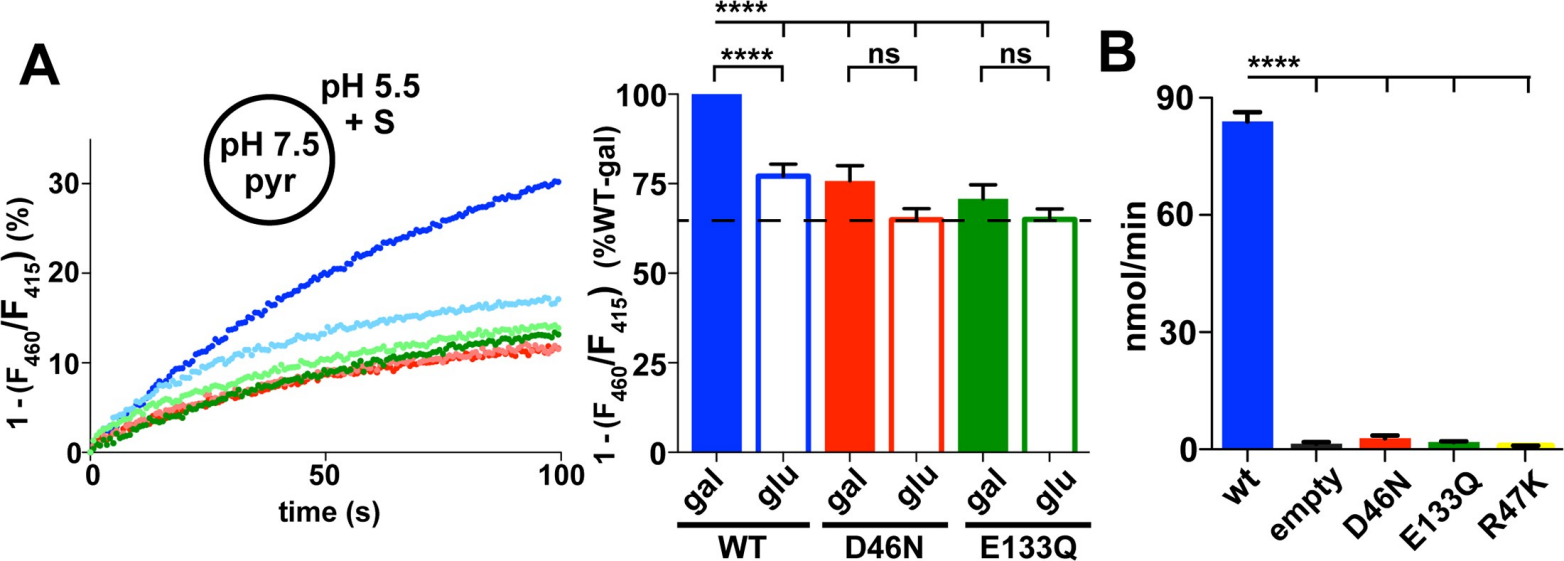
Functional residues coupled to proton or substrate transport. (A) Representative fluorescence of proteoliposomes reconstituted with WT (blue) or mutant DgoT (D460N red, E133Q green) in response to addition of D-gal (darker color) or glu (lighter color) at *t* = 0 seconds. Bar graph (right) shows the average change in fluorescence at *t* = 90 seconds. Filled bars indicate galactonate, open bars gluconate (*n* = 11). ***p < 0.001; ****p < 0.0001. (B) Whole-cell uptake of radiolabeled ^14^C-galactonate by WT and mutant DgoT exogenously expressed in an *E*. *coli* DgoT knock-out strain. WT but not D46N or E133Q DgoT confer ^14^C galactonate uptake (*n* = 3). The numerical data underlying this figure are included in S1 Data. DgoT, D-galactonate transporter; gal, galactonate; glu, gluconate; ns, not significant; pyr, pyranine; WT, wild type.

To test the functional significance of Asp46 and Glu133 for galactonate transport by DgoT, we replaced each separately by an uncharged residue (asparagine [D46N] and glutamine [E133Q], respectively) to mimic the electroneutrality of their protonated states. Reconstituting these purified proteins into liposomes, D46N and E133Q each reduce acidification by D-galactonate to the level observed with nontransported gluconate (Fig 3A).

**Fig 4:**
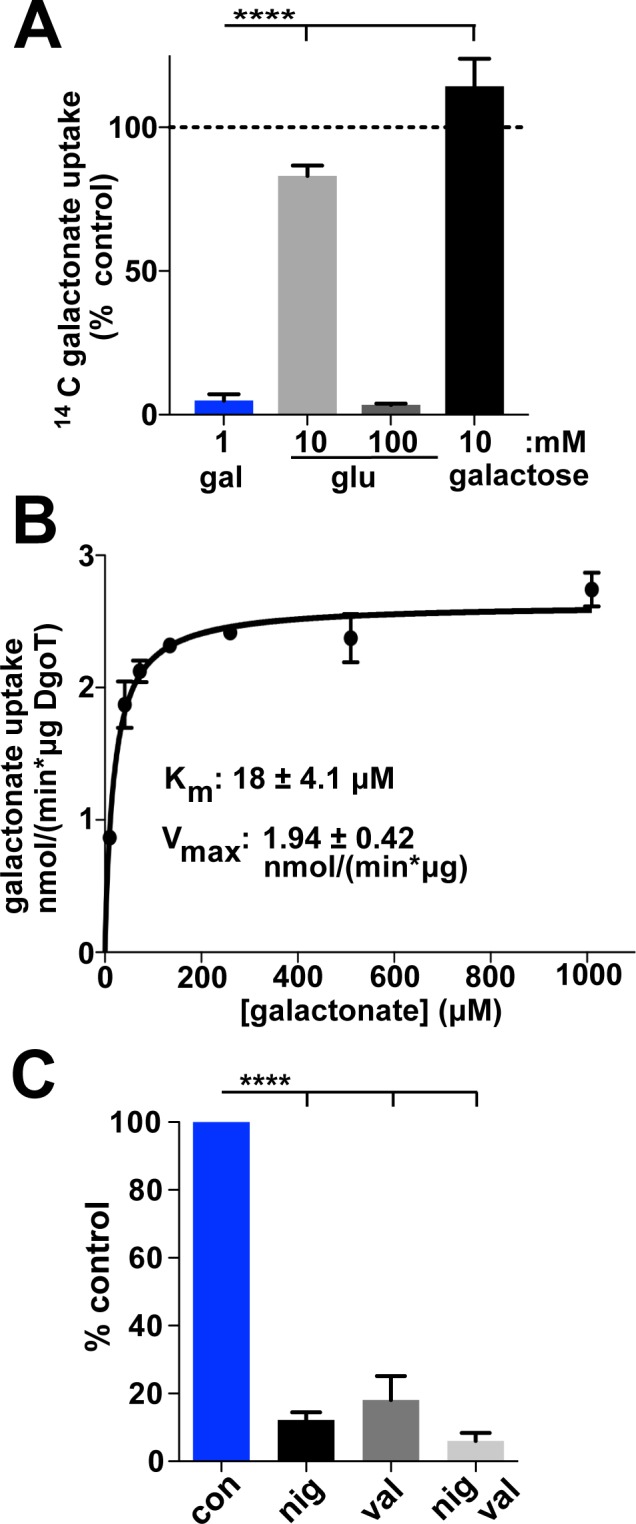
Properties of gal transport by DgoT. (A) Uptake of ^14^C-gal by *E*. *coli* expressing DgoT shows inhibition by 1 mM gal but not by 10 mM glu or galac (*n* = 3). (B) Representative concentration dependence of ^14^C-gal uptake by cells expressing WT DgoT. The data from multiple experiments were each fit to the Michaelis-Menten equation and used to calculate the mean Km = 18 ± 4.1 μM and Vmax = 1.94 ± 0.42 nmol/(min × μg) (*n* = 3). (C) *E*. *coli* spheroplasts expressing WT DgoT show inhibition of gal uptake by the H^+^ ionophore nig (dissipating ΔpH) and val (dissipating Δψ), demonstrating electrogenic cotransport of H^+^ and gal. The numerical data underlying this figure are included in S1 Data. ****p < 0.0001; con, control; DgoT, D-galactonate transporter; gal, galactonate; galac, galactose; glu, gluconate; Km, Michaelis-Menten constant; nig, nigericin; val, valinomycin; Vmax, maximal rate; WT, wild type.

To characterize galactonate transport by DgoT rather than rely on H^+^ flux to infer its properties, we developed a quantitative assay based on radiotracer uptake. Expressed in the *E*. *coli* DgoT mutant, WT DgoT confers robust uptake of ^14^C-galactonate relative to empty vector (Fig 3B). Using this assay, we reexamined the substrate specificity of DgoT by competition. Nonradioactive galactonate (1 mM) eliminates uptake of ^14^C-galactonate, whereas epimer gluconate or cyclized neutral sugar galactose (10 mM) has no effect, supporting the remarkable specificity for galactonate (Fig 4A). However, high levels (100 mM) of gluconate do eliminate uptake, indicating some recognition of the epimer. The Michaelis-Menten constant (Km) for galactonate is indeed 18 ± 4 μM (Fig 4B), approximately 100-fold lower than for glutamate transport by the VGLUTs (1–3 mM) [3, 15] and approximately 20-fold lower than for sialic acid transport by sialin (approximately 0.5 mM) [1, 2], tuning the Km values to the respective available substrate concentrations. To assess energy coupling by DgoT, we monitored ^14^C-galactonate uptake into spheroplasts. The H^+^ ionophore nigericin that dissipates the proton gradient ΔpH eliminates transport (Fig 4C). Dissipation of Δψ with the K^+^ ionophore valinomycin also eliminates galactonate uptake (Fig 4C), supporting the electrogenic nature of DgoT suggested by H^+^ flux measurements (Fig 1C).

Consistent with the absence of H^+^ flux, D46N and E133Q DgoT also accumulate very little ^14^C-galactonate in the radiotracer assay (Figs 3B and S8). Thus, active transport requires reversible protonation of both Asp46 and Glu133. The location of these residues at the end of the H^+^ tunnel from the periplasm further suggests specific roles in the H^+^ translocation pathway.

### Substrate binding stabilizes the occluded state

**Fig 5:**
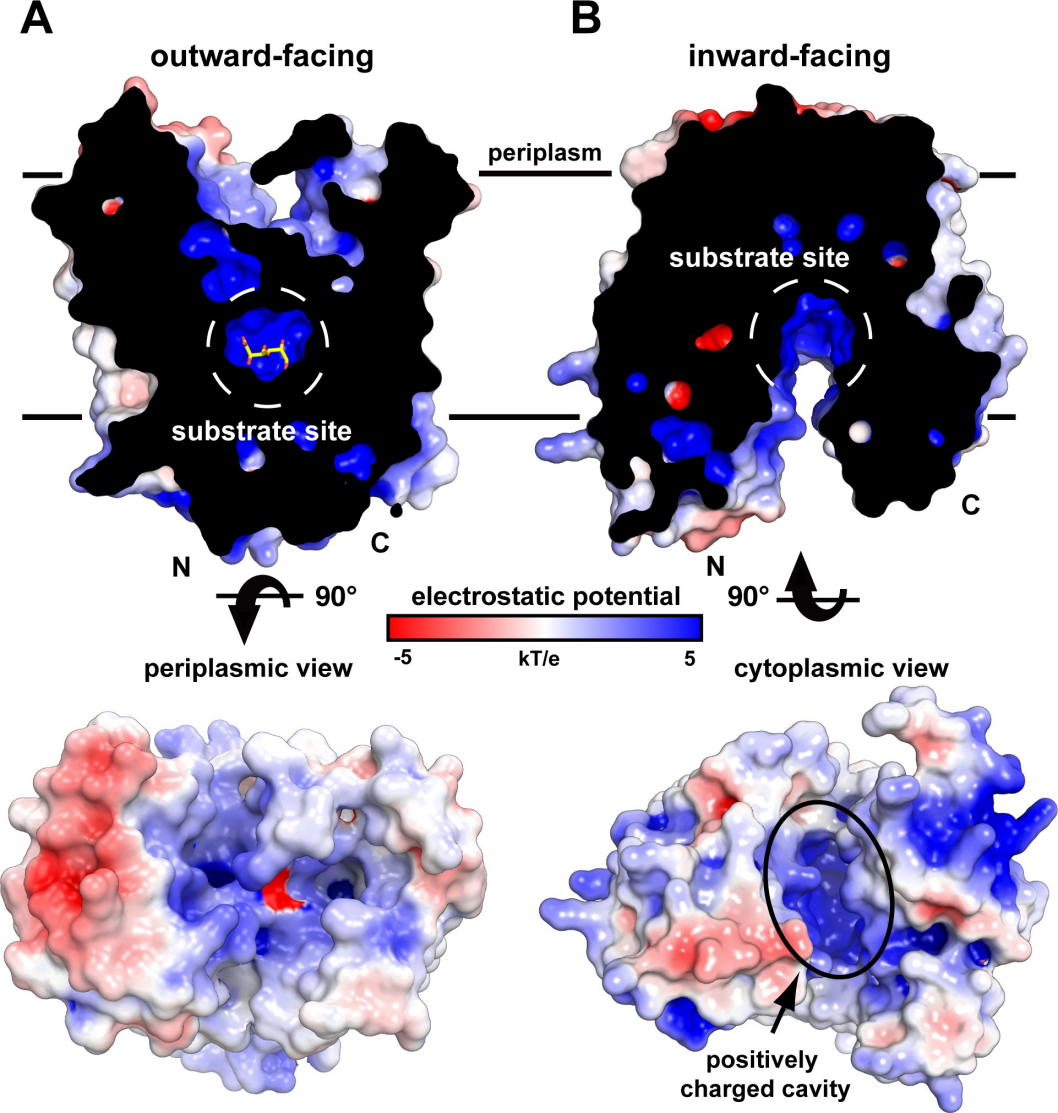
The structures of DgoT include substrate-bound outward-facing occluded and apo-, inward-facing open conformations. DgoT crystal structures shown in electrostatic surface potential representation. (A) A cross section through DgoT in the outward-facing occluded conformation shows galactonate (sticks) bound within the substrate recognition site (dashed white circle). The electrostatic potential of the periplasmic surface is shown below. (B) A cross section of DgoT in the inward-facing open conformation reveals an aqueous cavity with net positive surface charge. The electrostatic potential of the cytoplasmic surface is shown below. Both views highlight the positively charged binding site. DgoT, D-galactonate transporter.

The position of Glu133 at the end of the putative H^+^ tunnel, its importance for active transport, and its proximity to Arg47 facing the substrate cavity suggested that Glu133 might occupy a central role coupling H^+^ flux to transport of D-galactonate. We were able to crystallize E133Q DgoT with D-galactonate bound. At pH 9.0, this complex crystallized in an outward-facing conformation, in a different crystal form than for the WT protein alone, and refined to an R-factor/Rfree of 28.5%/30.0% at 3.5 Å resolution (Fig 5A and S1 Table). There are 2 independent copies of the protein in each asymmetric unit. This is the case in each of the 2 crystal forms; i.e., there are 2 independent structures of the molecule in each of the 2 molecular configurations. In each of the 2 major domains within each molecule, the congruence of intramolecular contacts and hydrogen bonds (i.e., 4 independent observations) provide strong cross-validation of intradomain interactions. As with the inward-open conformation, 2 essentially identical monomers (rmsd of 0.63 Å between their 345 Cα atoms) pack in pairs closely against each other sharing contacts from the lipophilic surface but in an antiparallel arrangement in the asymmetric unit (i.e., one molecule with its cytoplasmic side up packs against another with its cytoplasmic side down in a noncrystallographic 2-fold orientation). The structure includes residues 24 to 443 except for the 11–amino acid dynamic region in the cytoplasmic loop (residues 231–243) and 13–amino acid periplasmic end of TM7 (residues 276–291). In both conformations, the substrate cavity has a positive electrostatic surface potential to match the negatively charged substrate (Fig 5A and 5B). Computational modeling of glutamate at the position of Gln133 in the E133Q mutant structure predicts a pKa of approximately 10.1 [21]. Glu133 would thus be protonated in this conformation, supporting its possible role in proton transfer.

**Fig 6:**
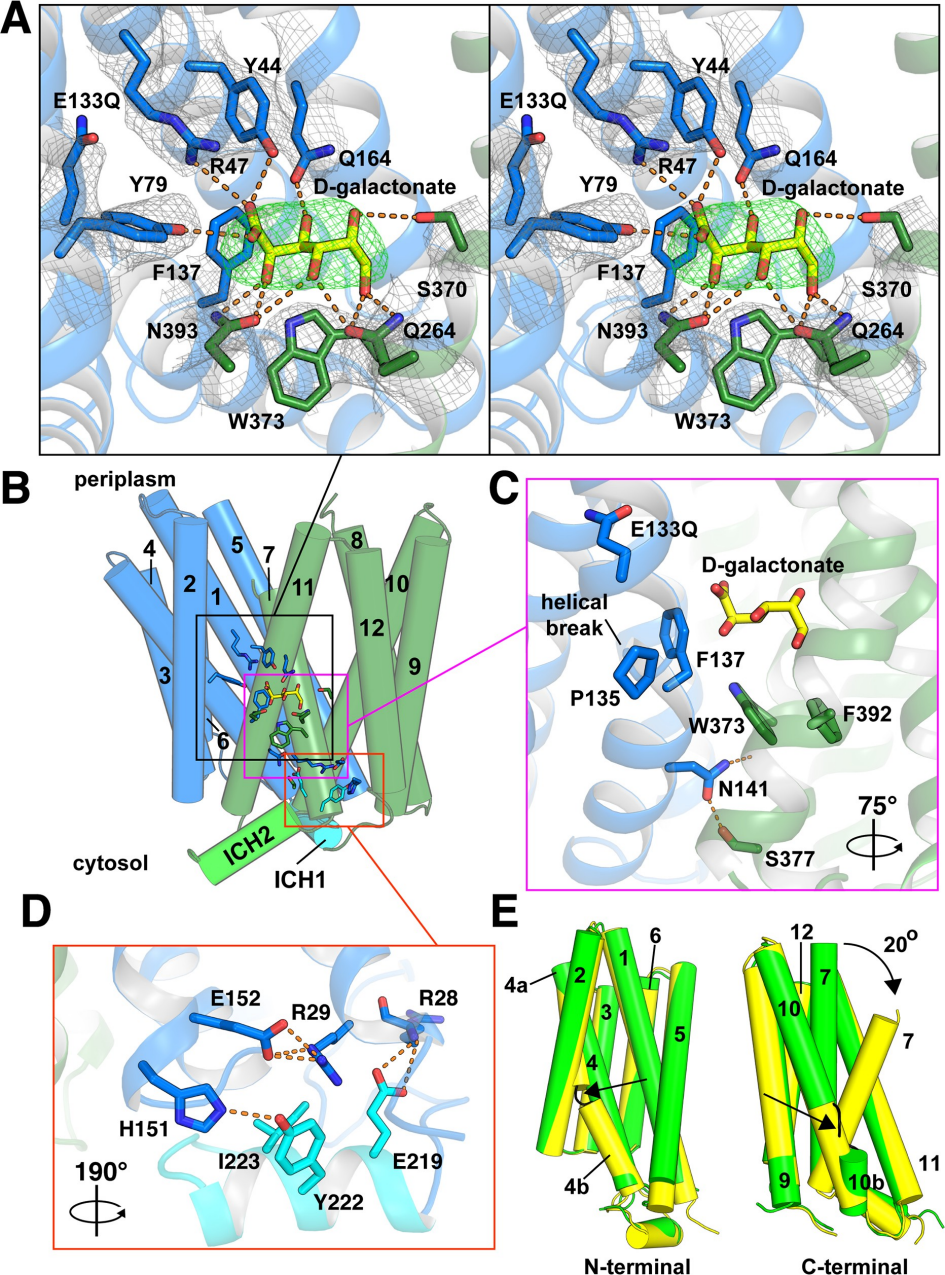
The outward-facing conformation contains D-galactonate occluded within the substrate recognition site. (A) Close-up stereo view of the substrate recognition site. Residues Q164, Q264, S370, and N393 form hydrogen bonds (orange dashes) with the 5 hydroxyl groups in D-galactonate. Y44 and Y79 form hydrogen bonds, and Arg47 forms a salt bridge with the carboxyl group of D-galactonate. The Fo-Fc density map of D-galactonate (green mesh) was contoured at 3σ, and the 2Fo-Fc density map (gray mesh) of DgoT residues was contoured at 1σ. (B) The overall structure of DgoT in cylinder representation defines the views shown in panel A (black rectangle), panel C (purple), and panel D (orange). N- and C-terminal domains are shown in blue and green, respectively. ICH1 is shown in cyan and ICH2 is shown in bright green. D-galactonate (yellow) is shown in stick representation. (C) Hydrophobic gating residues F137 and W373 interact with the substrate while forming contacts between N- and C-terminal domains. N141 forms a cytoplasmic gate by hydrogen bonding with the backbone carbonyl of W373 and the hydroxyl of S377. (D) Tripartite interactions between TM1, TM5, and ICH1. This view is rotated 190° as indicated for clarity. (E) Cylinder representation of the N- and C-terminal domains separated and rotated 90° (left) and 90° (right) show the extent of structural change between inward-open (green) and outward-occluded (yellow) states. With substrate bound, TM7 bends approximately 20° to partially occlude the substrate from the periplasmic side. The helical discontinuity of TM4 in the outward-occluded state and of TM10 in the inward-open state are indicated by black arrows. Arg47, arginine-47; DgoT, D-galactonate transporter; Fo-Fc, difference map; ICH, intracellular helix; TM, transmembrane.

In the outward-facing structure, Arg47 (TM1) forms a direct electrostatic interaction with the carboxyl group in D-galactonate through a 3.6 Å salt bridge (Fig 6). Arg47 is conserved in almost all other members of the SLC17 family from bacteria to mammals, consistent with this role. The 3.6 Å distance between the ε-NH_2_ of Arg47 in DgoT and the galactonate carboxyl suggests that the salt bridge is primarily electrostatic and may not involve hydrogen bonds, perhaps maintaining specificity without an affinity too high for rapid flux. In addition, conserved tyrosine-79 (Tyr79) coordinates the carboxyl group, suggesting a common role for these 2 residues in recognition of the carboxyl across the SLC17 family (Figs 6A and S3). Tyr44 in DgoT also coordinates the carboxyl group, although it is replaced by phenylalanine in the VGLUTs.

Four residues (Asn393, Gln264, serine-370 [Ser370], and Gln164) provide 8 hydrogen bonds with the 5 substrate −OH groups and confer high specificity (Fig 6A). The geometry of the stereogenic center C4-OH recognized by Asn393 in TM11 and the less conserved Gln264 in TM7 may account for the selectivity for galactonate over its epimer gluconate to compete with galactonate. The different geometry of gluconate in a solution may also contribute. In contrast to sugar transporters that generally segregate H^+^ and substrate translocation to different lobes, both N-terminal (Tyr 44 and Arg47 from TM1, Tyr79 from TM2, and Gln164 from TM5) and C-terminal domains (Gln264 from TM7, Ser370 from TM10, and Asn393 from TM11) of DgoT thus participate in substrate recognition.

### Conformational changes that couple alternating substrate access to proton transport

Comparison of the inward- and outward-facing structures defines conformational changes in DgoT that accompany substrate translocation (Figs 5 and 6). In addition to the rigid-body motions of N- and C-terminal lobes, TM7 bends inward over the substrate and provides a periplasmic gate for the outwardly oriented occluded state. Bending of TM7 has been identified in the structures of other MFS transporters (including D-xylose proton symporter [XylE], glucose transporter 3 [Glut3], melibiose permease [MelB], and lac permease [LacY]), suggesting a common mechanism for periplasmic gating [17, 22, 23]. Preventing access to the cytoplasmic side, TM4 and TM10 form major interhelical contacts in the outward-facing state. Aromatic residues Phe137 in TM4 and tryptophan-373 (Trp373) in TM10 both make hydrophobic interactions with the aliphatic surface of D-galactonate (Fig 6A and 6C). Removing the aromatic side chain in the equivalent phenylalanine residue of sialin increases the Km 15-fold (and the maximal rate (Vmax) 3-fold) for the physiological substrate glucuronic acid, with little effect on transport of sialic acid [24], supporting a role in substrate recognition and transport.

Comparing the 2 structures reveals hinges in TM4 and TM10 that accommodate changes associated with alternating access and substrate binding. In the outward-facing conformation, TM4 has a kink at Pro135, whereas TM10 is continuous (Fig 6C and 6E). In the inward-facing conformation, TM4 is continuous, whereas TM10 has a kink (Figs 6E and S5). In the outward-facing and substrate-bound state, Trp373 interacts with Ser377 (in TM10) and isoleucine-385 (Ile385) from TM11 (Fig 6C). Helical kinks are also seen in sugar transporters of the MFS [17, 19, 20], although in these proteins, the kinks occur within a single domain, perhaps because only that domain participates in substrate recognition.

Arg47 interacts with the substrate carboxyl in the outwardly oriented E133Q structure, but the residue occupies the same position relative to the rest of the N-terminal lobe as observed in the inwardly oriented state (S6 Fig). Protonation of Glu133 thus enables Arg47 to remain associated with D-galactonate without a major change in position of the TM1 arginine. However, the position of other residues that contact galactonate, especially F137 and W373, shows apparent movement between the 2 states (S5 and S6 Figs). In addition to the rotation in TM7, this movement in the substrate site closes the main cytoplasmic gate thereby occluding the substrate from both periplasmic and cytoplasmic sides.

The outwardly oriented structure lacks the putative H^+^ tunnel observed in the inwardly oriented state. The N- and C-terminal lobes that line the periplasmic entry site separate in the outwardly oriented structure, and the remaining tunnel is blocked (S7 Fig). However, it is plausible that closure of the tunnel secures protonation of the buried carboxyls as a key part of the cycle. The critical Glu133 has no clear access to the main substrate cavity in either structure (Figs 2 and 6), consistent with the existence of distinct pathways, separated in time and space, for galactonate and H^+^.

## Discussion

A critical question is how DgoT and sialin cotransport H^+^ with substrate, whereas the VGLUTs concentrate anion in a direction opposite the H^+^ electrochemical gradient. The 2 conformations of DgoT suggest a pathway for H^+^ translocation by the SLC17 family. The inward-facing WT structure has a tunnel capable of water-mediated H^+^ movement from the periplasmic space to a polar pocket in the N-terminal lobe. This putative H^+^ pathway terminates at Asp46, which may serve as a possible conduit for H^+^ to nearby Glu133, a residue highly conserved among SLC17 family members from bacteria to mammals, including the VGLUTs and sialin. Protons may access these acidic residues from the main vestibule rather than through the putative tunnel, but the crystal structures do not reveal an obvious alternate pathway. Because coupled ions in symporters generally access from the same side as the substrate, the presence of a H^+^ tunnel to the periplasm despite inward orientation to the cytoplasm makes DgoT unusual among solute carriers. Although we do not know the significance, this configuration could confer the potential for coupling to different steps of the transport cycle (e.g., reorientation of the empty carrier to the outside), allosteric activation, or even transport in different directions (as in the VGLUTs).

In both structures, Glu133 is sequestered from the solvent, so it is predicted to have a high pKa and be protonated. Mutation of either Asp46 (D46N) or Glu133 (E133Q) to mimic their neutral and/or protonated state eliminates active transport. Because sensitivity to valinomycin indicates that transport is electrogenic and suggests coupling to more than a single H^+^, Asp46 and Glu133 are candidates for transport of these protons. It is important to note that coupled to H^+^, DgoT concentrates substrate driven by a negative Δψ, in contrast to the positive Δψ that drives glutamate uniport by the VGLUTs.

We have not determined the stoichiometry of H^+^:D-galactonate transport by DgoT, but thermodynamic considerations help us to understand the number of protons that could be driven uphill by a galactonate gradient (Fig 1B and 1C), with implications for the concentration gradient of D-galactonate that could be produced in *E*.*coli*. The electrochemical gradient of protons across the membrane is composed of interconvertible electrical and chemical components according to Δµ_H+_ = Δψ − 2.3 (RT/F) ΔpH, where 2.3 RT/F is 58.8 mV at 20°C.

Moving a single charge across an electrochemical gradient E = 58.8 mV corresponds to *N*eE_m_ = FE_m_, approximately 1.4 kcal/mol of free energy. To move a proton uphill against ΔpH of 0.3 (Fig 1C), when Δψ = 0 (in the presence of valinomycin) thus requires 0.3 × 1.4 = +0.42 kcal/mol. Without valinomycin, transport is reduced as opposing Δψ develops due to excess positive charge, suggesting that the H^+^/D-galactonate stoichiometry is greater than 1 [26, 27]. A 10:1 gradient (out:in) of D-galactonate would provide a chemical-free energy of ΔG^0^_f_ = RT ln(C_in_/C_out_) = 1.4 log_10_(C_in_/C_out_) kcal/mole = −1.4 kcal/mol. Therefore, this gradient could pump approximately 2 to 3 H^+^ against the initial ΔpH of 0.3, consistent with electrogenic transport and the observed stoichiometry of >1. In *E*. *coli* at external pH of approximately 5.5, internal pH of approximately 7.5 (and hence ΔpH = 2), Δψ = −60 mV [28, 29], and electroneutral cotransport of a single proton by DgoT would contribute ΔG^0^_f_ of approximately −2.8 kcal/mol and produce a galactonate gradient of approximately 100:1. If the stoichiometry were 2 H^+^ per substrate, the resulting electrogenic transport would contribute ΔG^0^_f_ of approximately 7 kcal/mol to produce a galactonate gradient of approximately 10^5^:1.

At pH 7.5 outside, when ΔpH = 0 and Δμ_H+_ ≈ −100 mV, all due to Δψ [29], this would result in equilibration of galactonate (i.e., no gradient of galactonate) if driven by cotransport of a single H^+^ and hence no net charge movement. However, electrogenic transport driven by 2 H^+^ would contribute ΔG^0^_f_ of approximately 2.3 kcal/mol (due only to Δψ) to generate a gradient of approximately 50:1. Thus thermodynamic considerations help us to understand the physiological rationale for a H^+^ stoichiometry great than 1.

In the outward-open substrate-bound structure, Arg47 in DgoT interacts with the carboxyl on the anionic substrate and is highly conserved among other SLC17 members from bacteria to mammals. Two exceptions are the mammalian transporters SLC17A3 and SLC17A4, in which the equivalent residue is, respectively, asparagine and glutamine. These proteins transport organic anions, some of which (e.g., uric acid) do not contain a carboxyl group [30, 31], supporting a specific role for the TM1 arginine in DgoT and other SLC17 proteins in recognition of the substrate carboxyl [32, 33]. In total, 9 hydrogen bonds contribute directly to substrate recognition, of which 3 are donors to the carboxyl, and the remaining 6 can be acceptors to each of the substrate −OH groups, conferring the high specificity observed. In addition, both N- and C-terminal domains of DgoT participate in substrate recognition, whereas sugar transporters generally segregate H^+^ and substrate translocation to different lobes. Furthermore, comparison of the 2 structures shows that major conformational changes and local movement around the substrate site occur to accommodate substrate for transport.

**Fig 7:**
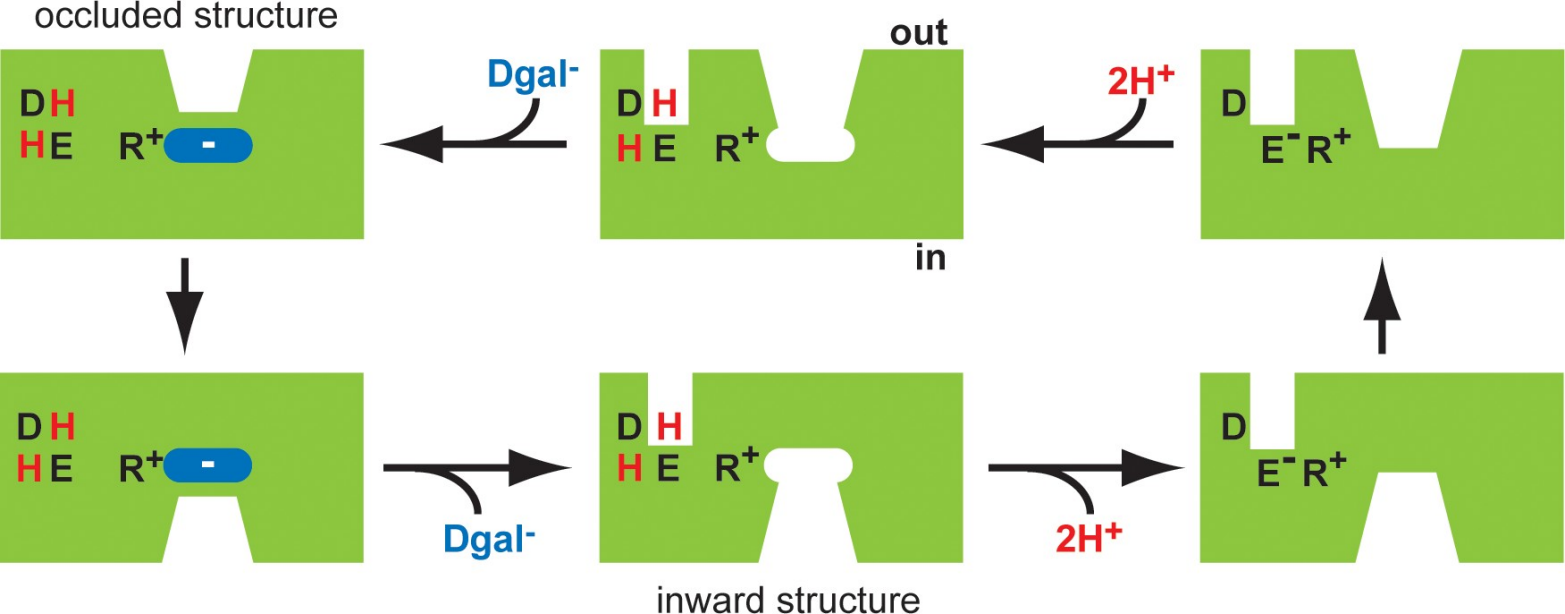
Proposed mechanism for coupling H^+^ to substrate translocation. Mechanism described counterclockwise from top left. In the outwardly oriented, occluded structure (top left), both Asp46 (D) and Glu133 (E) are protonated (H), liberating Arg47 (R) to interact with the anionic substrate (Dgal^−^) and transition from outward-facing to inward-facing conformation (bottom left). Release of substrate into the cytoplasm is captured in the inward-facing structure (bottom middle). Deprotonation of Glu133 could then enable the formation of a charge pair with Arg47 (bottom right) and so facilitate the transition of the empty transporter to the outward-facing state (top right). To account for electrogenic transport, we presume that Asp46 would also lose a H^+^ to the inside before this transition of the empty carrier. After reorientation to the outward-facing state, reprotonation of Asp46 and Glu133 (top middle) would allow Arg47 to interact with substrate (top left) and complete the cycle. We presume that the H^+^ tunnel to the periplasm occurs in the outward (as well as inward) orientation, before occlusion of the substrate, although we do not know whether proton(s) use this pathway or the main cavity to access the N-terminal polar pocket with Asp46 and Glu133. Arrows indicate the direction for inward uptake, although all the reactions are reversible. The VGLUTs lack an acidic residue in TM1 equivalent to Asp46 in DgoT and hence do not couple to H^+^. However, they contain a glu in TM4 equivalent to Glu133, and analogy with DgoT suggests that protonation of this site from the outside could allow the Arg in TM1 to bind neurotransmitter and facilitate transport. Protonation and deprotonation of a surrogate of Glu133 from the same side could account for allosteric activation of the VGLUTs by H^+^. This, in turn, could account for the efficient glu transport activity by synaptic vesicles, which is then inhibited by the higher pH in the synapse after vesicle fusion with the plasma membrane. Arg, arginine; Asp, aspartate; Dgal, D-galactonate; DgoT, D-galactonate transporter; Glu, glutamate; TM, transmembrane; VGLUT, vesicular glutamate transporter.

The 2 structures and the functional effects of mutations suggest a mechanism for coupling H^+^ to substrate translocation in the transport cycle. In the outward-facing conformation of DgoT, Glu133 is protonated. As a result, Arg47 can provide the positive charge required to complement the incoming substrate carboxyl (Fig 7) [34]. Sequestration of the substrate then stabilizes the neutral, protonated state of Glu133, consistent with the high pKa of the buried carboxyl inaccessible to the solvent. The transporter then reorients to the cytoplasmic side, where it releases substrate and H^+^. Similar to substrate, cytoplasmic release of H^+^ may occur from the substrate cavity, in contrast to the apparently specialized pathway from the periplasm. Glu133 must therefore deprotonate to enable movement of the empty transporter, a reduction in its pKa (by occlusion with water) accompanying formation of a charge pair with Arg47. Thus, Glu133 may release H^+^ via water to the side that closes when and only when Arg47 does not interact with substrate. After reorientation of the empty carrier from inside to out, another H^+^ enters, displacing the H^+^ bound at Asp46 to Glu133 so that external substrate can again bind.

The VGLUTs show conservation of Arg47, Arg126, and residues that contact galactonate in DgoT. The arginine in TM4 plays a critical role in glutamate transport by VGLUT [35] as well as in sialic acid transport by sialin [24]. Of the 2 key protonatable residues in DgoT (Asp46, Glu133), the VGLUTs (which are not driven by proton flux) contain only the highly conserved glutamate in TM4 (equivalent to Glu133 in DgoT) that is required for glutamate transport [36]. The VGLUTs thus retain the crucial H^+^ binding site of bacterial relatives (Glu133 in DgoT) and sialin. The VGLUTs function in an acidic environment, and protonation of Glu133 could be the titratable site for allosteric activation by H^+^ that we have previously observed [12]. Indeed, mammalian SLC17 proteins that operate at the plasma membrane (e.g., Na+-dependent phosphate transporter (NPT) 1–4) lack the conserved glutamate in TM4 and do not depend on low pH [30, 37]. These observations suggest that, very similar to their role in coupled transport, H^+^ may allosterically activate the VGLUTs by protonation of the TM4 glutamate for translocation of the loaded carrier and by deprotonation of the same site for reorientation of the empty carrier. The difference between the 2 mechanisms is that H^+^ are not coupled to glutamate transport, and VGLUTs indeed lack the Asp46 required for coupled transport by DgoT.

The structures also help us to understand the effect of mutations in the lysosomal H^+^ symporter sialin, which mediates efflux of sialic acid, a monosaccharide essential for oligosaccharide synthesis. Mutations in sialin cause a storage disease due to ineffective export and hence lysosomal accumulation of sialic acid [38]. The H183R mutation in human sialin prevents efflux of sialic acid, leading to infantile sialic acid storage disease (ISSD) [38, 39]. This mutation eliminates transport activity with no effect on sialin expression or localization [1, 2]. DgoT contains an asparagine at the equivalent position in TM4, and this residue (Asn141) forms hydrogen bonds with TM10 through the hydroxyl group of Ser377 and the backbone carbonyl of Trp373, thereby positioning Trp373 to interact with the substrate directly (Figs 6B, 6C and S5). Thus, the H183R mutation in sialin would impair cytoplasmic closure of the aqueous cavity and so inhibit outward opening of sialin, accounting for the loss of transport activity and the severe clinical phenotype.

Sialin and the VGLUTs (but not the sugar transporters) also contain sequences corresponding to DgoT ICH1 and, to a slightly lesser extent, ICH2. The R39C mutation in sialin causes a milder sialic acid storage disorder, Salla disease [38, 39]. In contrast to H183R, R39C reduces but does not eliminate transport [1, 2]. The corresponding residue Arg29 in DgoT lies at the cytoplasmic end of TM1, surrounded by conserved residues in TM5 and ICH1 (Fig 6D). It forms a salt bridge with Glu152 in TM5. Thus, the R29C mutation in DgoT would disrupt interactions between TM1, TM5, and ICH1 (Fig 6D). Conserved residues Glu219 and Tyr222 in the ICH1 domain form salt bridges with, respectively, Arg28 (TM1) and His151 (loop between TM4 and TM5). ICH1 thus provides an alternative mechanism to connect TM1 and TM5. As a result, the R39C mutation in sialin might be expected to impair but not eliminate the tripartite interactions between TM1, TM5, and ICH1, accounting for the Salla disease phenotype.

In summary, the 2 structures of DgoT suggest a common mechanism for divergent ionic coupling by the SLC17 family: protonation of Glu133 effectively releases Arg47 to bind and translocate substrate. If there is no substrate bound, Glu133 must give up its H^+^ so that it can form a charge pair with Arg47 and reorient empty to complete the transport cycle. Thus, translocation of the conjoint substrate site—loaded with substrate or empty—mandates neutralization of Arg47 and Glu133, either by binding of substrate to Arg47 and protonation of Glu133 or by formation of a charge pair between deprotonated, charged Glu133 and Arg47.

## Materials and methods

### Cloning and expression

The full-length *E*. *coli* DgoT gene (accession number AKK15832.2) was subcloned into pQE60 (Qiagen, Venlo, Netherlands) with a C-terminal modified decahistidine tag preceded by a thrombin cleavage site. Mutant constructs were generated from this plasmid by site-directed mutagenesis and confirmed by sequence analysis. The protein was produced in 2 different ways: first for crystallization in the lipidic cubic phase (LCP) and second for crystallization by vapor diffusion.

### DgoT purification for vapor diffusion crystallization

For WT crystals, *E*. *coli* C41 cells, transformed with pQE60 DgoT WT were grown at 37°C in TB medium supplemented with 2 mM MgSO_4_. At a cell density of 0.6 to 0.8 OD_600_, the temperature was reduced to 18°C, and the culture was induced with 0.5 to 1 mM IPTG, incubated at 18°C, and harvested after 16 hours. A typical yield of 200 g from 6 L culture was split into 50 g aliquots and stored at −80°C. Harvested cells (50 g) were resuspended in 20 mM Tris (pH 7.4), 300 mM NaCl (250 mL) containing 1× protease inhibitors (Sigma, Burlington, MA) and lysed using the Emulsiflex C3 homogenizer (ATA Scientific, Australia) for 5 to 6 cycles at 15,000 to 20,000 psi. The whole cell lysate was centrifuged at 10,000 or 18,000*g* for 20 minutes to remove debris, and the supernatant was centrifuged at 185,500*g* for 1 hour to collect the membranes. The membranes were split into 3.5 g aliquots, flash frozen in liquid nitrogen, and stored at −80°C until further use.

A 3.5 g aliquot of membranes was resuspended in 50 ml 20 mM Tris (pH 7.4), 150 mM NaCl, 10% glycerol using a glass Dounce homogenizer. The resuspended membranes were solubilized by adding 200 mg n-dodecyl-β-maltoside (DDM; Anatrace, Maumee, OH) per gram membrane, for a final DDM concentration of approximately 1.4% (w/v). The membranes were solubilized by stirring at 4°C for 2 hours, the insoluble material removed by sedimentation at 185,500*g* for 20 minutes, and the supernatant incubated with 5 ml Talon cobalt affinity resin (Clontech) at 4°C for 2 hours under gentle nutation. After drainage, the resin was washed with 10 column volumes of 20 mM Tris (pH 7.4), 150 mM NaCl, 10% glycerol, 0.05% β-DDM, 10 mM imidazole (wash buffer), and 2 column volumes of wash buffer supplemented with 500 mM NaCl. The protein was eluted with 4 column volumes of wash buffer containing 150 mM imidazole (elution buffer). Imidazole was removed from the eluate with a 10-DG desalting column (Bio-rad, Hercules, CA), and the polyhistidine tag was removed by digestion overnight at 4°C with α-thrombin. The digested protein was concentrated to 0.5 ml using a 100 kDa molecular weight cut-off (MWCO) centrifuge concentrator (Millipore) followed by filtration through 0.2 μm PVDF. To change the detergent from β-DDM to β-NG (Anatrace), 0.5 ml protein was injected onto a size exclusion column (TSK3000; Tosch Bioscience) pre-equilibrated with 10 mM HEPES (pH 7), 150 mM NaCl, 10% glycerol, 0.5 mM TCEP, and 0.2% β-NG (SEC buffer). Peak fractions were pooled and concentrated for crystallization using a 50 kDa MWCO centrifuge concentrator (Millipore, Hayward, CA).

For DgoT E133Q, protein expression and purification were performed as described for WT DgoT, with several exceptions. During protein purification, the protein-bound affinity resin was washed with 10 column volumes of 20 mM Tris (pH 7.4), 150 mM NaCl, 10% glycerol, 0.05% β-DDM, and 10 mM imidazole (wash buffer) followed by 2 column volumes of wash buffer supplemented with 25 mM imidazole and 500 mM NaCl. For both protein concentration steps, a 50 kDa MWCO centrifuge concentrator (Millipore) was used instead of the 100 kDa MWCO concentrator at the first step.

### Crystallization, lipidic environment, and data collection

For crystallization of WT DgoT, protein was relipidated into *E*. *coli* polar lipids (Anatrace) according to the High Lipid-Detergent (HiLiDe) method [40] using 2.33:1 protein:lipid and 5:1 detergent:lipid weight ratios. For a typical HiLiDe trial, 50 μL DgoT (5 mg/ml) was used. HiLiDe-treated DgoT was crystallized via hanging drop methods in a 96-well crystallization plate (Greiner) using the TTP mosquito (TTP Labtech). For hanging drops, 150 nl 5 mg/ml protein was mixed with 150-nl–well solution containing 39% PEG 400 and 100 mM sodium acetate (pH 5.35) and grown at 4°C. Initially, small, rod-shaped crystals grew and disappeared within 2 to 3 days, possibly reflecting sensitivity to precipitant concentration that occurs through vapor diffusion. To grow larger and more stable crystals, 50 μl of Al’s oil (Hampton Research, Viejo, CA) was placed over the well solution to slow vapor diffusion. Also, DgoT was pre-incubated with 10 mM sodium gluconate (Sigma) on ice for 10 minutes prior to crystallization set-up. Crystals were harvested and flash frozen in the well solution using liquid nitrogen. X-ray diffraction data sets were collected at the Advanced Light Source Beamline 8.3.1. Data were collected at a wavelength of 1.1159 Å.

For crystallization of DgoT E133Q, protein was also crystallized via hanging drop but remained unlipidated because HiLiDe was not performed. DgoT E133Q was pre-incubated with 10 mM Na galactonate for 10 minutes on ice prior to crystallization; 100 nl 7 to 8 mg/ml protein was mixed with 100-nl–well solution containing 32% PEG 1000 and 100 mM glycine (pH 9) and grown at 4°C. Rectangular crystals appeared around 3 to 4 weeks and continued to grow over 9 months to about 150 to 200 μm in length. Crystals were harvested and flash frozen in the well solution using liquid nitrogen. X-ray diffraction data sets were collected at the Advanced Light Source Beamline 8.3.1. Data were collected at a wavelength of 1.1158 Å.

### Data processing and structure determination

For the inward-facing (WT DgoT) crystals, data sets were recorded on a MAR CCD detector, processed, integrated, and scaled using the HKL2000 package [41]. The space group was P2_1_ (*n* = 2). Because diffraction was anisotropic, the data were submitted to the UCLA anisotropy server [42] for ellipsoidal truncation. This removes reflections with F/σ < 3. The best data set was truncated to 2.9 × 3.8 × 3.8 Å resolution along the a, b, and c axis, respectively. After ellipsoidal truncation and anisotropic scaling, B-factor sharpening was applied using a negative isotropic B-factor of −68 Å^2^. The resolution for reporting data and refinement statistics (S1 Table) is based on the statistically significant correlation coefficient value of CC_1/2_ [43]. If resolution cut-off is based on the criterion of <I/σ(I)> *>* 1 the resolution is reduced to 3.5 Å. However, the quality of the electron density maps shows details in places as in a 2.9 Å density map.

For E133Q crystals, data sets were recorded on a Pilatus3 detector, and because this involved 3,600 images, the XDS package is more appropriate for the processing, integration, and scaling [44]. The space group was C2 (*n* = 2). Post-processing for anisotropic data was not necessary. These crystals diffracted isotropically but to a more limited highest resolution of 3.5 Å. This resolution limit was determined by statistical significance of the CC_1/2_ criterion [43]. Despite the low <I/σ(I)> = 0.2 and the low CC_1/2_ (0.16) data in the outer shell, it is still statistically significant. If resolution cut-off is based on the criterion of <I/σ(I)> *>*1, the resolution would be reduced to 4.0 Å. The quality of the map shows details as in a 3.5 Å map.

Both DgoT structures were determined by MR using Phaser-MR (PHENIX). For the inward-facing structure, phases were determined by Phaser-MR (PHENIX) using a poly-alanine model of the crystal structure of GlpT [16] (PDB ID: 1PW4) that is 24% sequence identical as a search model. The unit cell volume indicated the presence of 2 molecules in the asymmetric unit, with a solvent content of 70%. Initial phases yielded an electron density map that identified 12 TM helices suggesting the secondary structure of MFS transporters. Reiterative model building in COOT [45] followed by refinement runs in phenix.refine (PHENIX) [46] programs were used. For initial refinement strategies, rigid-body refinement [47], individual B-factor refinement, simulated-annealing refinement (torsion: 2,500 K start; 300 K final; 25 steps), and translation libration screw (TLS) rotation model refinement were used. PHENIX-autobuild was used in calculating iterative-build composite omit maps followed by intermittent simulated-annealing composite omit maps to reduce model bias during building [46, 48, 49]. Once Rfree reached approximately 32%, hydrogens were added to the model and were refined using individual B-factor refinement with X-ray and/or stereochemistry target weight optimization. The final model of DgoT includes residues 10 to 219 and 229 to 427 and has an R_free_ value of 29.7%.

For the outward-facing E133Q structure, the N-terminal and C-terminal domains of the WT DgoT structure were separately used as search models for MR. Two molecules in the asymmetric unit were identified similar to WT. Initial phases yielded an electron density map that identified the 12 TM helices and the 2 ICHs, but in a different structural arrangement than WT, suggesting a different conformation. Initial refinement was performed using TLS and restrained refinement with 50 to 100 cycles of jelly-body refinement implemented in Refmac [50] along with ProSMART [51] to produce general fragment-based restraints for low-resolution refinement. Once Rfree reached approximately 35%, additional refinement under PHENIX was done using rigid-body refinement [47], individual B-factor refinement, and TLS refinement. D-galactonate assignment was guided using 2Fo-Fc and Fo-Fc maps. All structural figures were generated using PyMol (Schrödinger, New York, NY). Composite SA omit map (−100 Å2) in Fig 4A was B-factor sharpened (PHENIX). Electrostatic potential surfaces were calculated using APBS [52]. The proton tunnel was visualized using Mole [53].

### Sodium D-galactonate preparation

To our knowledge, the only commercially available form of D-galactonate is the insoluble calcium salt. We therefore used previously reported methods to produce the Na^+^ salt from Ca^2+^ D-galactonate [54, 55]. Briefly, 10 g Ca^2+^ D-galactonate (City Chemical, West Haven, CT) was resuspended in 100 ml water. An equimolar amount of oxalic acid dihydrate (2.93 g; Sigma, Burlington, MA) was added to the mixture and stirred for 10 minutes at 55°C. The precipitated calcium oxalate was then separated from aqueous D-galactonic acid by filtration under vacuum through 0.22 μm nylon. Sodium hydroxide was titrated into the solution to pH 7. Absolute ethanol was added in a 3:1 (v/v) ratio, the mixture stored at 4°C overnight, and the resulting crystalline precipitate removed by filtration and washed with absolute ethanol. The precipitate was dried for 24 hours in a vacuum desiccator. The resulting crystalline Na^+^ D-galactonate was then stored at room temperature for subsequent use. The crystalline precipitate was analyzed by ^1^H-NMR using a Bruker 400 mHz Avance III HD spectrometer, confirming the purity of Na^+^ D-galactonate.

### DgoT reconstitution and transport assay

Purified DgoT was reconstituted using preformed vesicles permeabilized with Triton X-100 [56]. Briefly, 10 mg *E*. *coli* polar lipids (Avanti Polar Lipids, Alabaster, AL) were dissolved in 1 ml chloroform, dried first under nitrogen and then under vacuum overnight. The dried lipid film was rehydrated in 1 ml 0.5 mM HEPES (pH 7.5), 150 mM N-methyl-D-glucamine (NMDG)-methanesulfonate (a large organic cation that should not permeate), 2 mM MgSO_4_, and 0.5 mM pyranine (reconstitution buffer) by incubating at 37°C for 30 minutes and resuspended through pipetting. To form unilamellar vesicles, the lipid suspension was subjected to 4 cycles of freeze-thaw, alternating between liquid nitrogen and 25°C, with sonication in a water bath for 5 minutes between freeze-thaw cycles. The lipids were then extruded through a 200 or 400 nm filter, sonicated for 5 minutes, and finally extruded through a 100 nm filter. The extrusions both involved 20 passages through each filter.

To prepare proteoliposomes, the liposomes were destabilized by adding 0.6% (v/v) Triton X-100 and incubated at 4°C overnight under gentle nutation. Purified DgoT was added to the destabilized liposomes at a 1:30 protein:lipid (w:w) ratio and incubated for 1 hour at 4°C. To extract detergent, SM2 Bio-beads (Bio-rad, Hercules, CA) were added sequentially in 4 steps. First, 200 mg SM2 Bio-beads were added and incubated for 1 hour at 4°C. This addition was repeated once (step 2) followed by the addition of 400 mg SM2 Bio-beads for an additional 2 hours (step 3). For the final step, 600 mg SM2 Bio-beads were added and incubated overnight. To remove unincorporated pyranine and the Bio-beads, the proteoliposome mixture was separated using a 10-DG desalting column (Bio-rad, Hercules, CA), eluting with 4 ml pyranine-free reconstitution buffer according to the manufacturer’s protocol.

To measure transport by DgoT, an inwardly directed H^+^ gradient was produced by adding 200 μl proteoliposomes into a cuvette containing 1.35 ml 0.5 mM MES (pH 5.5), 150 mM NMDG methanesulfonate, and 5 mM MgSO_4_ (reaction buffer). Transport was initiated by adding 50 μl D-galactonate or D-gluconate in reaction buffer, and uptake was monitored at 30°C by measuring the fluorescence emission at 510 nm upon dual excitation at 415 nm (F1) and 460 nm (F2). The fluorescence data were collected at 0.7-second intervals using a F-4500 fluorimeter (Hitachi). The ratio of fluorescence emission at the 2 excitation wavelengths (F2/F1) was normalized to the initial ratio (F2_o_/F1_o_) and the results plotted as 1 − [(F2/F1)/(F2_o_/F1_o_)] (as a percentage), with galactonate added at *t* = 0. To assess coupling between galactonate and H^+^, we used the same conditions but with lumenal pH 7.2 and external pH 7.5 (both buffered with Hepes rather than MES), 5 mM K^+^ in both lumenal and external solutions, with or without 0.2 μM valinomycin. The fluorescence measurements at the low pH were performed in triplicate on multiple occasions. The fluorescence measurements at the higher pH were performed using 2 reconstitutions, and the initial rates were determined by subtracting the drift in fluorescence before galactonate addition from the time points after. Subtracted data were fitted to the equation y = A + B(x) + Ce^−D(x)^, which contains a single exponential decay component [Ce^−D(x)^] plus a linear term [A + B(x)] that better fits the rate of decay in liposome controls.

### Cell-based radiotracer uptake

DgoT-deficient *E*. *coli* strain dgoT727(del)::kan (Keio collection; *E*. *Coli* Genetic Stock Center, Yale, New Haven, CT) was lysogenized with IPTG-inducible T7 RNA polymerase using the λDE3 kit (Novagen Madison, WI). DgoT variants were subcloned into pQE60 vector containing a C-terminal deca-histidine tag and transformed into the lysogenic strain. One colony was picked to inoculate a 150 ml LB culture (+100 μg/mL carbenicillin and 35 μg/ml kanamycin) and grown at 37°C with shaking until the OD_600_ reached 1.0. Basal protein expression from the T7 promoter was sufficient for uptake assays, and no IPTG was added during growth. After harvesting the cells at 3,800*g* using a table-top centrifuge, the pellet was washed twice with 5 mM MES (pH 6.5), 150 mM KCl (MK buffer), and resuspended to an OD_600_ of 2.0. To measure substrate transport, 150 μl cell suspension was pre-incubated at 25°C for 5 minutes; 50 μl MK solution containing 10 μM ^14^C-galactonate (American Radiolabeled Chemicals) was added to initiate the reaction, the mixture was incubated 30 seconds or 2 minutes at 25°C, the reaction was terminated by filtration through 0.45 μm nitrocellulose (Millipore), and the filters were washed 3 times with 2 ml cold MK buffer before measurement of the bound radioactivity by scintillation counting. Incubation for 30 seconds was used for competition and saturation experiments, and 2 minutes was used for the comparison of different DgoT constructs. All transport assays were performed in triplicate on at least 3 independent cell preparations.

The expression of DgoT protein was monitored by immunoblotting with a polyhistidine antibody conjugated to horseradish peroxidase (HRP; Qiagen, Venlo, Netherlands) and the chemiluminescent detection of HRP monitored with a ChemiDoc MP imaging system (Bio-rad, Hercules, CA). The absolute amounts of WT and mutant DgoT expression were estimated by comparing the band intensities of each to a serial dilution of purified DgoT (100 to 3 ng) on the same western blot.

### Spheroplast uptake

Spheroplast generation was adapted from previously established methods [57]. DgoT constructs in pQE60 vector were transformed into the *E*. *coli* DgoT knock-out strain (see previous section). One colony was picked to inoculate a 150 ml LB culture (+100 μg/mL carbenicillin and 35 μg/mL kanamycin) and grown at 37°C with shaking overnight. The overnight culture (3 ml) was used to inoculate a 150 ml LB culture (+carbenicillin/kanamycin) and grown at 37°C with shaking until the OD_600_ reached 1.0. At that point, 25 ml culture was sedimented at 3,800 rpm for 10 minutes using a table-top centrifuge, the pellet was washed twice in 30 mM Tris (pH 8; 16 ml per 0.2 g cell pellet) and was resuspended in 30 mM Tris (pH 8) with 20% sucrose (approximately 80 ml buffer per g cell pellet), lysozyme was added (to 20 μg/m), and the mixture was incubated for 5 to 10 minutes. After 1:1 dilution with 30 mM Tris (pH 8) buffer, EDTA was added (to 1 mM), and the resulting spheroplasts were incubated for 10 to 15 minutes with gentle nutation. The spheroplasts were then sedimented at 16,000*g* for 20 minutes and resuspended in 5 mM MES (pH 5.5), 150 mM KCl, and 20% (w/v) sucrose (MKS-5.5 buffer) supplemented with 20 mM glycerol and 5 mM MgCl_2_ to an OD_600_ of 2.0.

To measure net flux, 150 μl spheroplast solution was pre-incubated at 25°C for 5 minutes, 50 μl MKS-5.5 with 10 μM ^14^C-galactonate was added, and the mixture was incubated for 2 minutes. After incubation, the spheroplasts were subjected to filtration through 0.45 μm nitrocellulose and were washed 3 times with 2 ml cold MK buffer before measurement of the bound radioactivity by scintillation counting. For the analysis of ionophores, 150 mM KCl was replaced by K acetate in both internal and external solutions.

To measure exchange, the spheroplasts were preloaded with 1 mM nonradioactive galactonate by incubation overnight at 4°C, sedimented at 16,000*g*, washed 3 times with 10 ml MKS-5.5, and resuspended in MKS-5.5 containing 20 mM glycerol to an OD_600_ of approximately 1.1. Transport was measured as previously described for net uptake by spheroplasts, with or without nigericin or valinomycin (both at 2 μM). As described above, both net flux and exchange were measured in triplicate using at least 3 independent preparations. We subtracted the value for empty vector from WT and mutant DgoT and normalized the exchange rate by mutants for their reduced expression relative to WT (S8 Fig).

## Data analysis and statistics

All graphs and statistics from the functional data were calculated using GraphPad Prism software. The length of each bar represents the mean, and errors bars represent the standard error of the mean. Statistical significance was determined by one-way ANOVA with Bonferroni post hoc comparison.

[25]

## Supporting information

S1 Fig Purified recombinant DgoT behaves as a monodispersed protein by size exclusion chromatography and reconstitutes into proteoliposomes. (A) Size exclusion chromatography (TSK 3000) and Coomassie stain of purified *Escherichia coli* DgoT separated by SDS-polyacrylamide gel electrophoresis. The major elution peak along fractions 24 to 30 corresponds to monomeric DgoT with an apparent weight of approximately 35 kDa. (B) Silver stain of individual fractions after sucrose gradient fractionation (5%–60%) of DgoT proteoliposomes. Proteoliposomes containing reconstituted DgoT appear in fractions F7 to F9. Aggregated and/or nonincorporated DgoT appears at the bottom of the gradient in F12. Purified DgoT (lane 2) was added for molecular weight comparison. (C) Traces (left) show representative fluorescence ratios of proteoliposomes containing pyranine with (left) and without DgoT (right), both formed at pH 7.2, in external solution at pH 7.5, with or without 0.2 µM valinomycin. Yo is the ratio recorded at the addition of 10 mM D-galactonate, and Yf is the ratio recorded at the end of the recording. Both internal and external solutions contained 5 mM K^+^. (D) Statistical analysis of the results from 9 DgoT proteoliposome and 4 liposome experiments fitted to the equation Y = A + B(x) + C^−D(x)^ that contains both linear and exponential terms to account for the proton leak (linear) and galactonate-driven H^+^ flux (exponential). A is the y-intercept, B the linear slope between Yo and Yf, C the difference between Yo and Yf, and D the rate of exponential decay. The curve fitting is shown in C. Best-fit values of each component are listed along with regression (R^2^). DgoT, D-galactonate transporter.
(TIF)

S2 Fig The asymmetric crystallographic unit contains two polypeptide chains. (Top) Cylinder representations of the 2 polypeptide chains in the inward-facing conformation of DgoT. β-NG (magenta) appears in the aqueous cavity of both chain A (A) and chain B (B) but at different locations in each. Chain A also contains a molecule of gluconate (yellow). (Bottom) Cross section of the corresponding electrostatic surface potential representations for chains A (A) and B (B). (C) Stick representation of residues interacting with gluconate and the nearby β-NG molecule. (D) Closest interatomic distances between β-NG and gluconate shown in angstroms. (E) Close-up of β-NG located within the aqueous cavity of chain B. Electron density map for C through E shown as 2Fo-Fc map contoured at 1σ. β-NG, β-Nonylglucoside; DgoT, D-galactonate transporter; Fo-Fc, observed structure factor–calculated structure factor.
(TIF)

S3 Fig Sequence alignment of DgoT with other SLC17 family members. Sequence alignment of *E*. *coli* DgoT (accession number: AKK15832.2), human VGLUT1 (SLC17a7, accession number: Q9P2U7.1), human VGLUT2 (SLC17a6, accession number Q9P2U8.1), human VGLUT3 (SLC17a8, accession number Q8NDX2.1), and human sialin (SLC17a5, accession number Q9NRA2.2). Amino acid numbering above corresponds to *E*. *coli* DgoT. Strongly conserved residues are highlighted in a red box with white text; Asp31 in *E*. *coli* DgoT and other less well conserved but important residues are highlighted in a purple box with black text. The secondary structure (above) includes 12 TM domains and 2 ICHs, with the outward-occluded helices shown above and the inward-open below. N- and C-terminal helices are highlighted in blue and green, respectively. Substrate-binding residues are labeled with an asterisk (*). Asp31, Aspartate-31; DgoT, D-galactonate transporter; ICH, intracellular helix; SLC17, solute carrier 17; TM, transmembrane domain; VGLUT, vesicular glutamate transporter.
(TIF)

S4 Fig A proton tunnel connects the periplasm to the N-terminal polar pocket. (A) DgoT viewed from the periplasm is shown in electrostatic surface potential representation, with arrow indicating the entrance to the putative H^+^ tunnel. Acidic residues including E59 and E180 contribute to the strong negatively charged electrostatic surface. (B) Cross section through electrostatic surface potential representation, viewed from the plane of the membrane. The entrance (near E180) has a diameter of 3.6 Å, a constriction (near Pro279 and Thr297) of 2.4 Å, and an exit (near Asp46) of 5.0 Å. Asp46, Aspartate-46; DgoT, D-galactonate; Pro279, Proline-279; Thr297, Threonine-297.
(TIF)

S5 Fig Helix 10 becomes discontinuous in the inward-open conformation. (A) TM10 separates from TM4 in the inward-facing conformation (colored), breaking the helix in TM10 but re-establishing the continuity of TM4 that was disrupted in the outward-facing conformation (gray) (B) For each panel, the N- and C-terminal domains were aligned from the other structure for comparison. 2Fo-Fc density map (1σ) of residues from TM4 and TM10 from the inward-facing conformation (C) and outward-facing conformation (D). Fo-Fc, observed structure factor–calculated structure factor; TM, transmembrane domain.
(TIF)

S6 Fig Relative movement of residues at the substrate binding site. (A) View of the substrate binding site showing residues and substrate. Alignment of N- and C-terminal domains from the inward-facing conformation (gray) onto the outward-facing conformation (colored). (B) Close-up of the alignment between Arg47 (TM1) and glutamate (inward-facing) or glutamine (outward-facing) at position 133. (C) A composite, simulated annealing “omit map” (1σ) is shown in a close-up stereo view of the substrate recognition site. Residues Gln164, Gln264, Ser370, and Asn393 form hydrogen bonds (orange dashes) with the 5 hydroxyl groups in D-galactonate. Tyr44 and Tyr79 form hydrogen bonds, and Arg47 forms a salt bridge with the carboxyl group of D-galactonate. Arg, Arginine; Asn, Asparagine; Gln, Glutamine; Ser, Serine; TM, transmembrane domain; Tyr, Tyrosine.
(TIF)

S7 Fig The proton tunnel collapses when switching to the outward-facing conformation. (Left panel) Cross section of inward-facing DgoT viewed from the periplasm shown in surface representation (N terminal: blue; C terminal: green). Arrow indicates the entrance to the putative H^+^ tunnel, ending at Asp46. (Right panel) Cross section of the N-terminal domain in the outward-facing conformation shows the remnant of the H^+^ tunnel with an occlusion. Asp46, Aspartate-46; DgoT, D-galactonate transporter.
(TIF)

S8 Fig Expression of DgoT mutants relative to WT. (A) Immunoblot of extracts from DgoT^−^ *E*. *coli* transformed with WT *E*. *coli* DgoT, empty pQE60 vector, D46N, E133Q, or the double D46N/E133Q DgoT detected using a polyhistidine antibody and chemiluminescence. Standards of WT purified *E*. *coli* DgoT are shown to the right. (B) Immunoblot of extracts from DgoT^−^ *E*.*coli* transformed with empty pQE60 vector, WT, and R47K DgoT. DgoT, D-galactonate transporter; WT, wild type.
(TIF)

S1 Table Data collection and refinement statistics (MR). MR, molecular replacement.
(DOCX)

S1 Data Data underlying Figs 1B, 1C, 3A, 3B, 4A, 4B and 4C. (XLSX)

## Abbreviations

Arg47: arginine-47
Asn141: asparagine-141
Asp46: aspartate-46
β-NG: β-nonylglucoside
DDM: n-dodecyl-β-maltoside
DgoT: D-galactonate transporter
Fo-Fc: observed structure factor—calculated structure factor
Gln179: glutamine-179
GlpT: Glycerol-3-phosphate transporter
Glut3: glucose transporter 3
HiLiDe: High Lipid-Detergent
HRP: horseradish peroxidase
ICH: intracellular helix
Ile385: isoleucine-385
ISSD: infantile sialic acid storage disease
Km: Michaelis-Menten constant
LacY: lac permease
LCP: lipidic cubic phase
MelB: melibiose permease
MFS: major facilitator superfamily
MR: molecular replacement
MWCO: molecular weight cut-off
NMDG: N-methyl-D-glucamine
NPT: Na^+^-dependent phosphate transporter 1 (SLC17A1)
Phe278: phenylalanine-278
Pro279: proline-279
rmsd: root-mean-square deviation
Ser370: serine-370
SLC17: solute carrier 17
Thr172: threonine-172
TLS: translation libration screw
TM: transmembrane domain
Trp373: tryptophan-373
Tyr79: tyrosine-79
VGLUT: vesicular glutamate transporter
Vmax: maximal rate
WT: wild type
XylE: D-xylose proton symporter
