# Supplementary figures and images for: Structures suggest a mechanism for energy coupling by a family of organic anion transporters
Source: PLoS Biol. 2019 May 13;17(5):e3000260. doi: 10.1371/journal.pbio.3000260 (PMC6532931; doi:10.1371/journal.pbio.3000260)

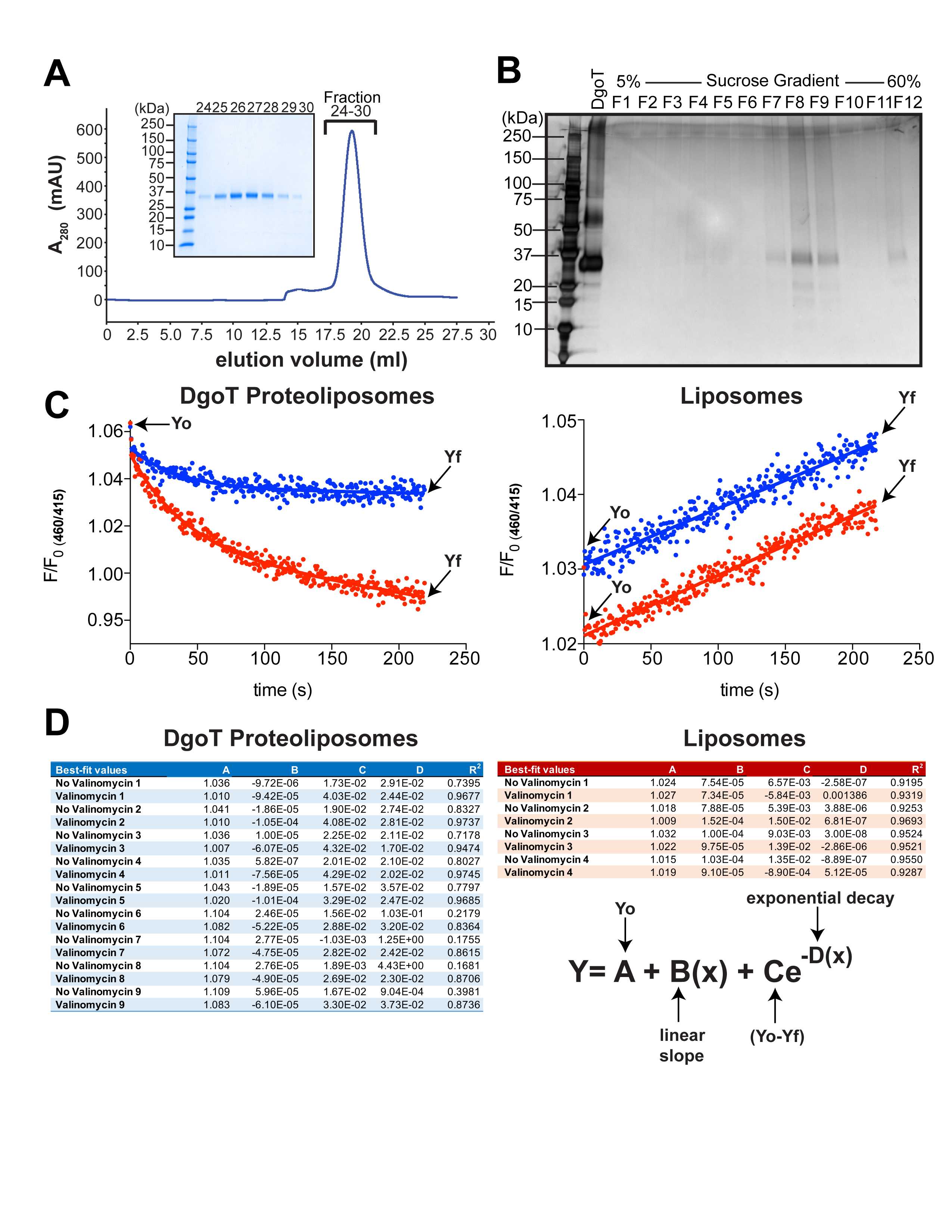

Supplement: S1 Fig — (A) Size exclusion chromatography (TSK 3000) and Coomassie stain of purified Escherichia coli DgoT separated by SDS-polyacrylamide gel electrophoresis. The major elution peak along fractions 24 to 30 corresponds to monomeric DgoT with an apparent weight of approximately 35 kDa. (B) Silver stain of individual fractions after sucrose gradient fractionation (5%–60%) of DgoT proteoliposomes. Proteoliposomes containing reconstituted DgoT appear in fractions F7 to F9. Aggregated and/or nonincorporated DgoT appears at the bottom of the gradient in F12. Purified DgoT (lane 2) was added for molecular weight comparison. (C) Traces (left) show representative fluorescence ratios of proteoliposomes containing pyranine with (left) and without DgoT (right), both formed at pH 7.2, in external solution at pH 7.5, with or without 0.2 µM valinomycin. Yo is the ratio recorded at the addition of 10 mM D-galactonate, and Yf is the ratio recorded at the end of the recording. Both internal and external solutions contained 5 mM K+. (D) Statistical analysis of the results from 9 DgoT proteoliposome and 4 liposome experiments fitted to the equation Y = A + B(x) + C−D(x) that contains both linear and exponential terms to account for the proton leak (linear) and galactonate-driven H+ flux (exponential). A is the y-intercept, B the linear slope between Yo and Yf, C the difference between Yo and Yf, and D the rate of exponential decay. The curve fitting is shown in C. Best-fit values of each component are listed along with regression (R2). DgoT, D-galactonate transporter. (TIF) [file pbio.3000260.s001.tif]

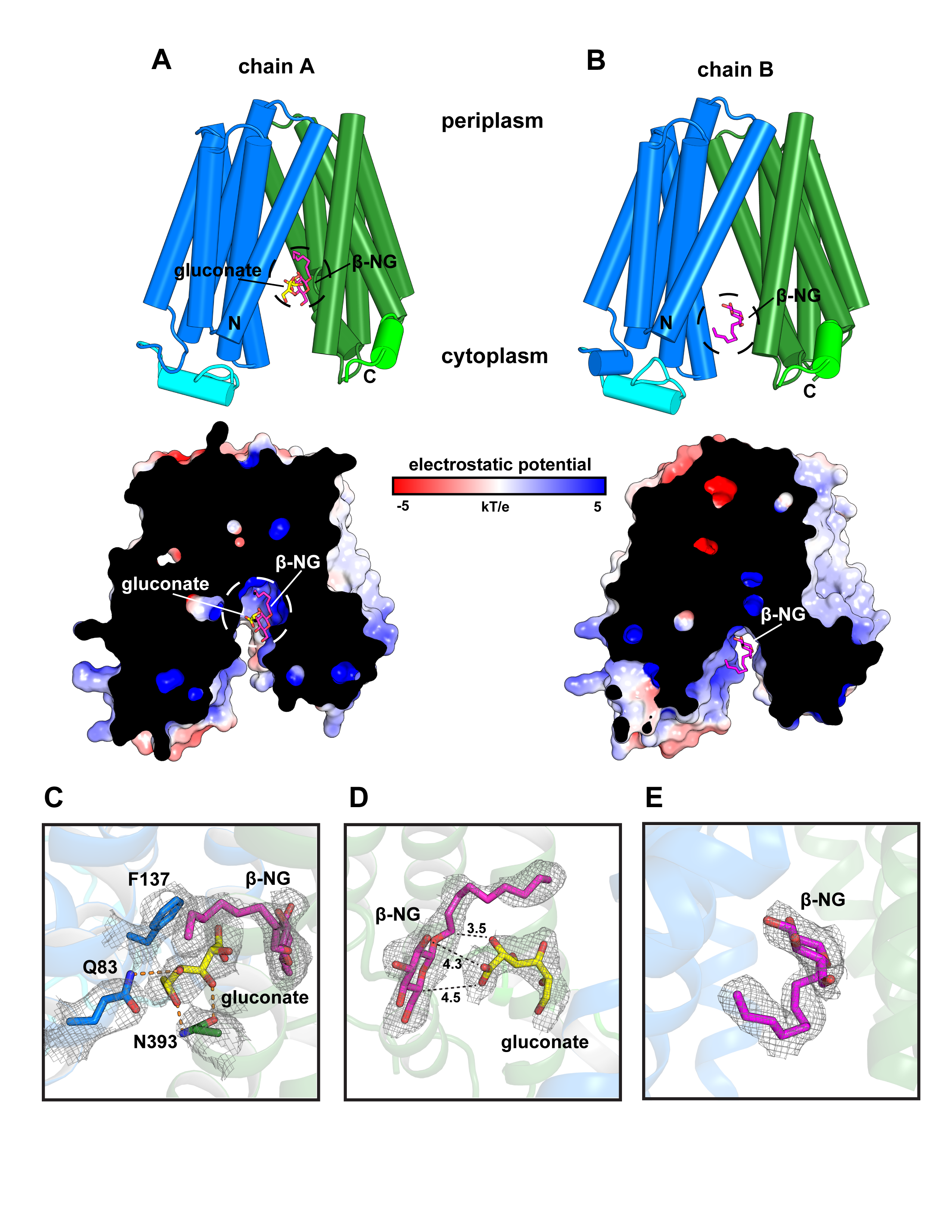

Supplement: S2 Fig — (Top) Cylinder representations of the 2 polypeptide chains in the inward-facing conformation of DgoT. β-NG (magenta) appears in the aqueous cavity of both chain A (A) and chain B (B) but at different locations in each. Chain A also contains a molecule of gluconate (yellow). (Bottom) Cross section of the corresponding electrostatic surface potential representations for chains A (A) and B (B). (C) Stick representation of residues interacting with gluconate and the nearby β-NG molecule. (D) Closest interatomic distances between β-NG and gluconate shown in angstroms. (E) Close-up of β-NG located within the aqueous cavity of chain B. Electron density map for C through E shown as 2Fo-Fc map contoured at 1σ. β-NG, β-Nonylglucoside; DgoT, D-galactonate transporter; Fo-Fc, observed structure factor–calculated structure factor. (TIF) [file pbio.3000260.s002.tif]

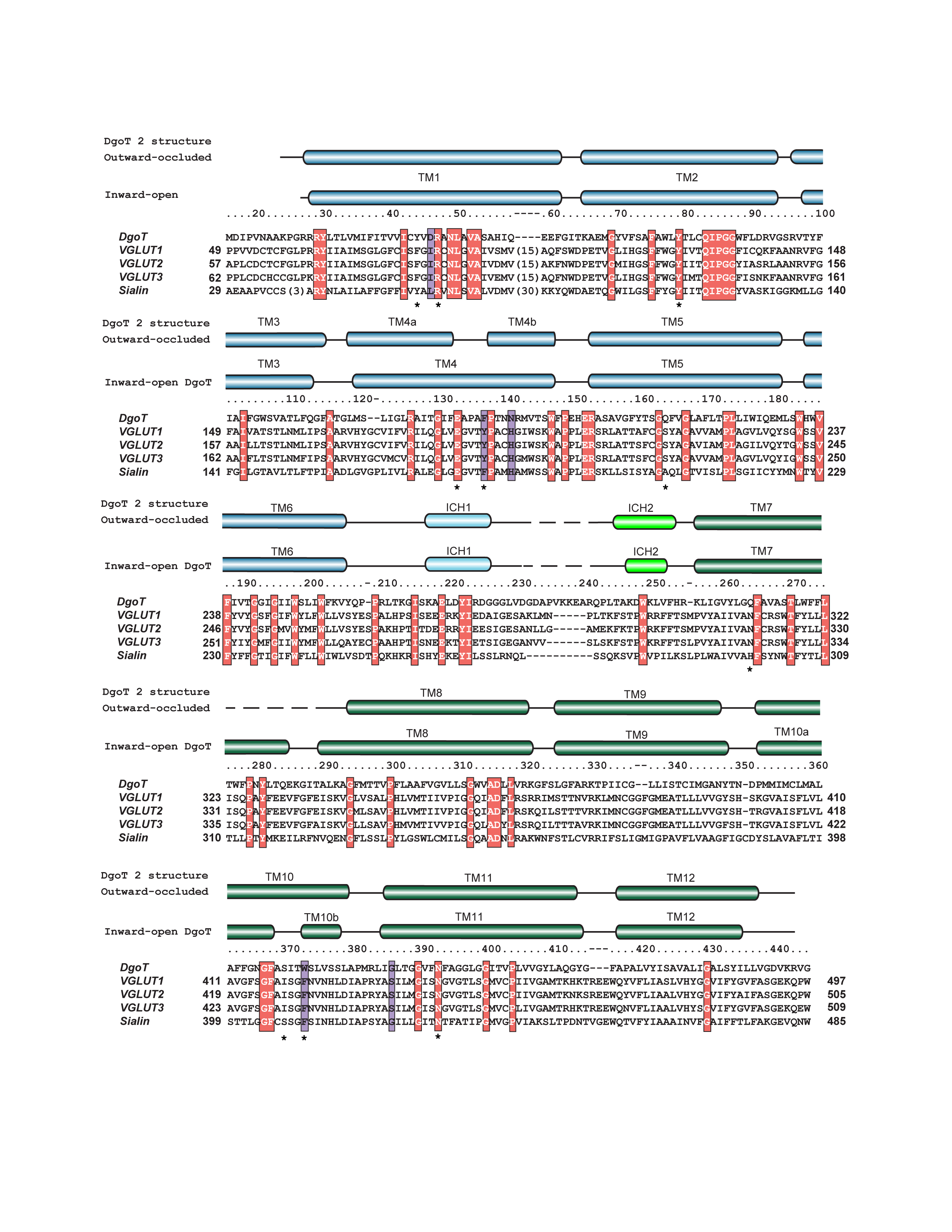

Supplement: S3 Fig — Sequence alignment of E. coli DgoT (accession number: AKK15832.2), human VGLUT1 (SLC17a7, accession number: Q9P2U7.1), human VGLUT2 (SLC17a6, accession number Q9P2U8.1), human VGLUT3 (SLC17a8, accession number Q8NDX2.1), and human sialin (SLC17a5, accession number Q9NRA2.2). Amino acid numbering above corresponds to E. coli DgoT. Strongly conserved residues are highlighted in a red box with white text; Asp31 in E. coli DgoT and other less well conserved but important residues are highlighted in a purple box with black text. The secondary structure (above) includes 12 TM domains and 2 ICHs, with the outward-occluded helices shown above and the inward-open below. N- and C-terminal helices are highlighted in blue and green, respectively. Substrate-binding residues are labeled with an asterisk (*). Asp31, Aspartate-31; DgoT, D-galactonate transporter; ICH, intracellular helix; SLC17, solute carrier 17; TM, transmembrane domain; VGLUT, vesicular glutamate transporter. (TIF) [file pbio.3000260.s003.tif]

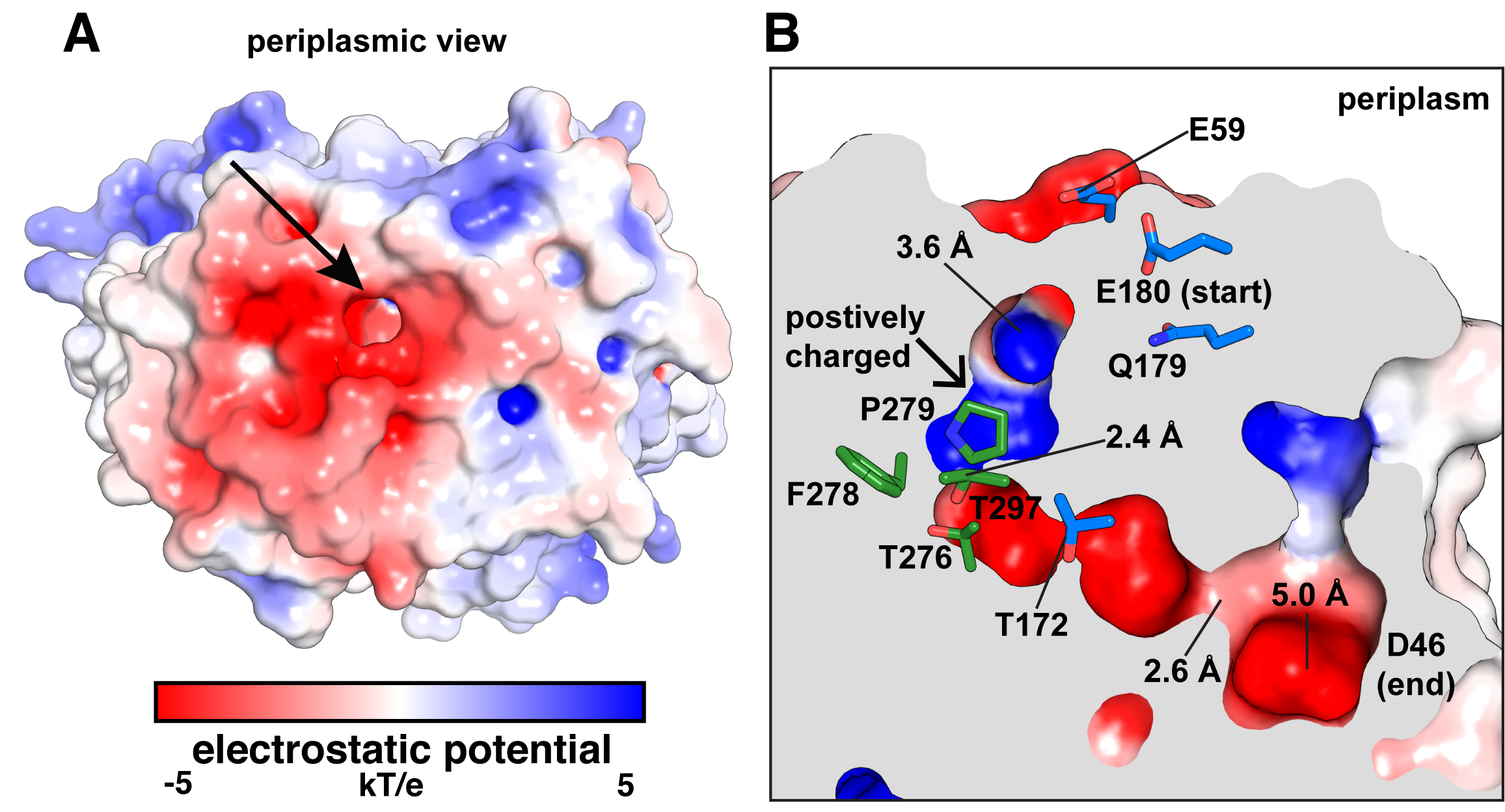

Supplement: S4 Fig — (A) DgoT viewed from the periplasm is shown in electrostatic surface potential representation, with arrow indicating the entrance to the putative H+ tunnel. Acidic residues including E59 and E180 contribute to the strong negatively charged electrostatic surface. (B) Cross section through electrostatic surface potential representation, viewed from the plane of the membrane. The entrance (near E180) has a diameter of 3.6 Å, a constriction (near Pro279 and Thr297) of 2.4 Å, and an exit (near Asp46) of 5.0 Å. Asp46, Aspartate-46; DgoT, D-galactonate; Pro279, Proline-279; Thr297, Threonine-297. (TIF) [file pbio.3000260.s004.tif]

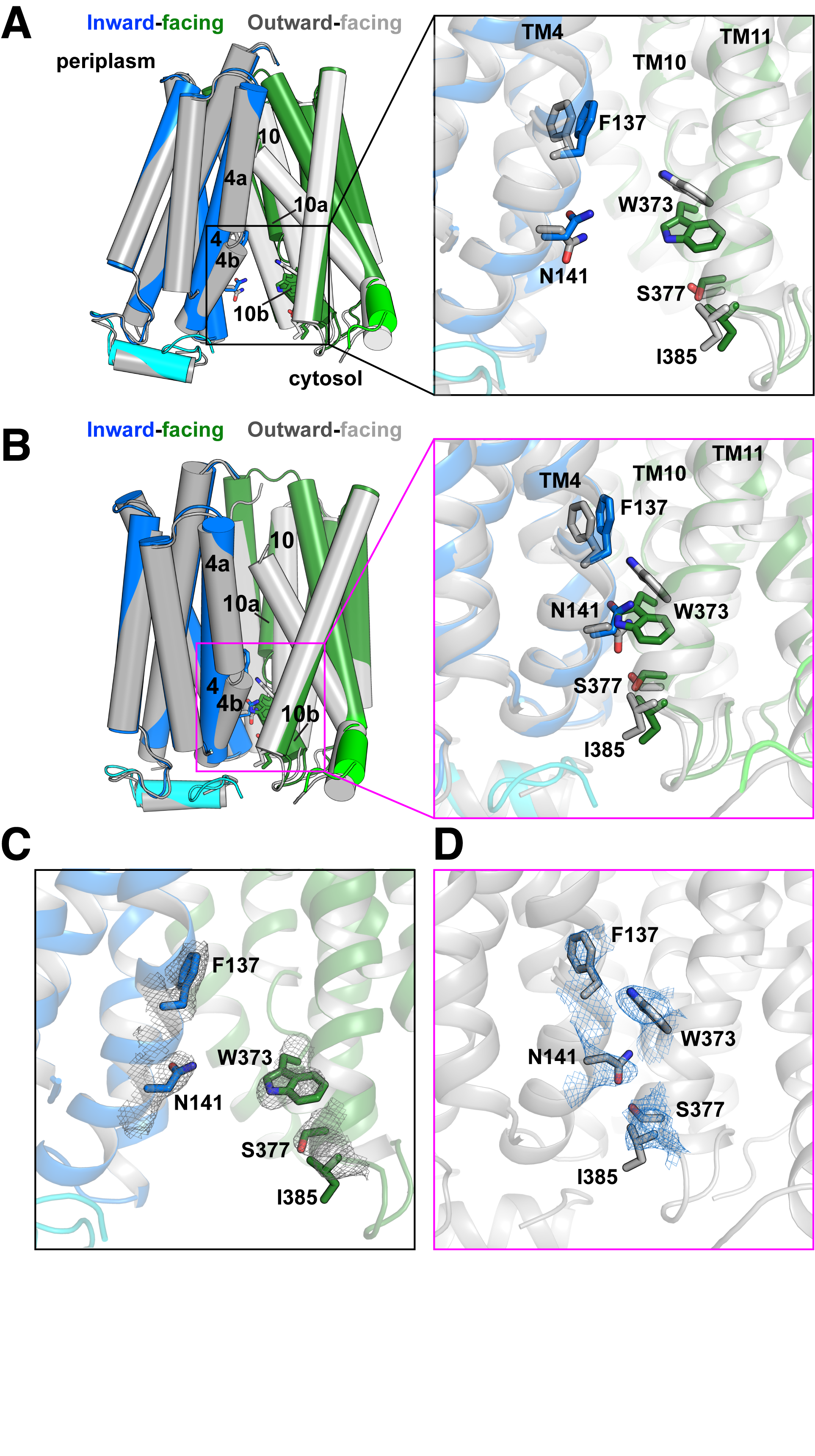

Supplement: S5 Fig — (A) TM10 separates from TM4 in the inward-facing conformation (colored), breaking the helix in TM10 but re-establishing the continuity of TM4 that was disrupted in the outward-facing conformation (gray) (B) For each panel, the N- and C-terminal domains were aligned from the other structure for comparison. 2Fo-Fc density map (1σ) of residues from TM4 and TM10 from the inward-facing conformation (C) and outward-facing conformation (D). Fo-Fc, observed structure factor–calculated structure factor; TM, transmembrane domain. (TIF) [file pbio.3000260.s005.tif]

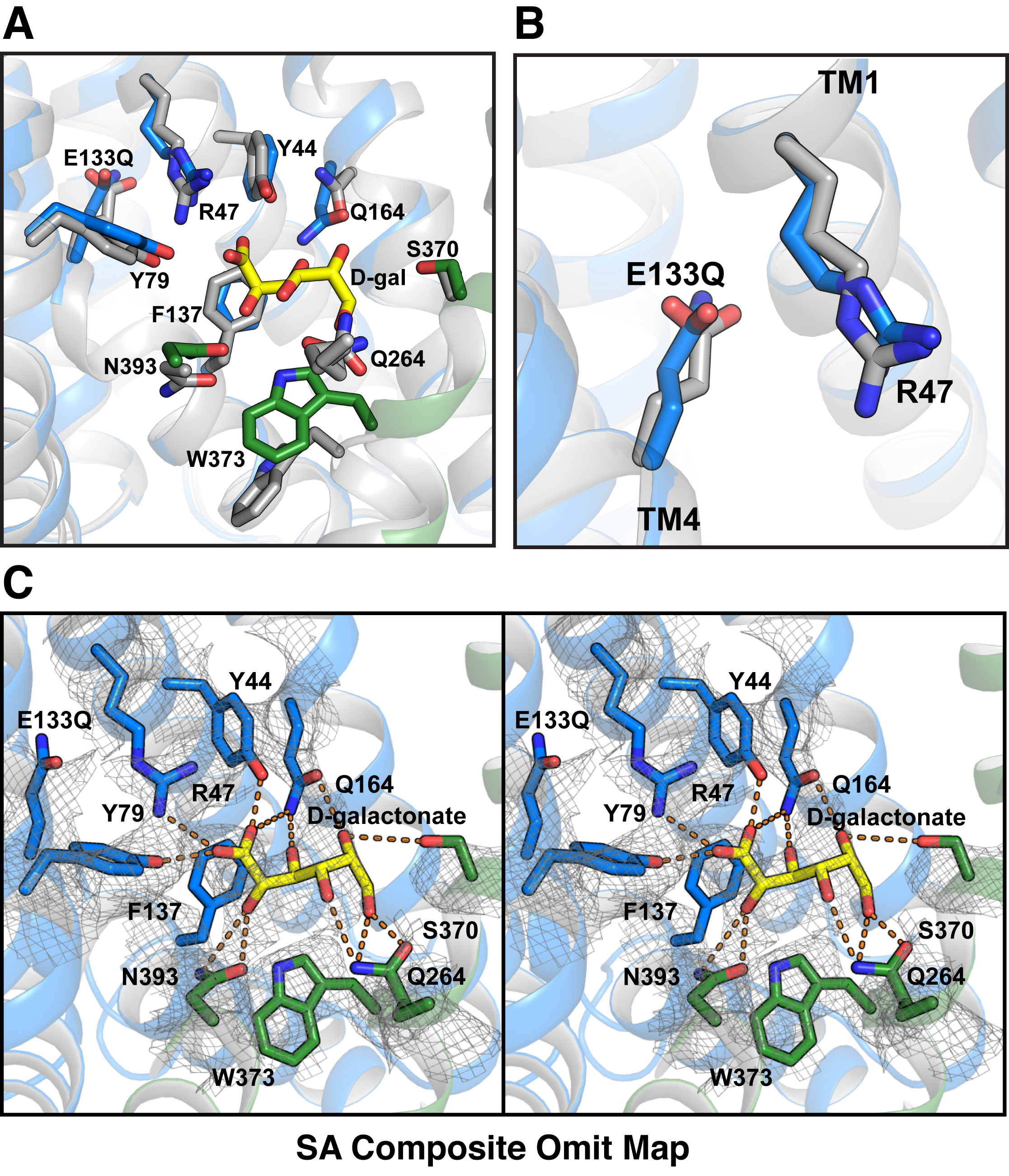

Supplement: S6 Fig — (A) View of the substrate binding site showing residues and substrate. Alignment of N- and C-terminal domains from the inward-facing conformation (gray) onto the outward-facing conformation (colored). (B) Close-up of the alignment between Arg47 (TM1) and glutamate (inward-facing) or glutamine (outward-facing) at position 133. (C) A composite, simulated annealing “omit map” (1σ) is shown in a close-up stereo view of the substrate recognition site. Residues Gln164, Gln264, Ser370, and Asn393 form hydrogen bonds (orange dashes) with the 5 hydroxyl groups in D-galactonate. Tyr44 and Tyr79 form hydrogen bonds, and Arg47 forms a salt bridge with the carboxyl group of D-galactonate. Arg, Arginine; Asn, Asparagine; Gln, Glutamine; Ser, Serine; TM, transmembrane domain; Tyr, Tyrosine. (TIF) [file pbio.3000260.s006.tif]

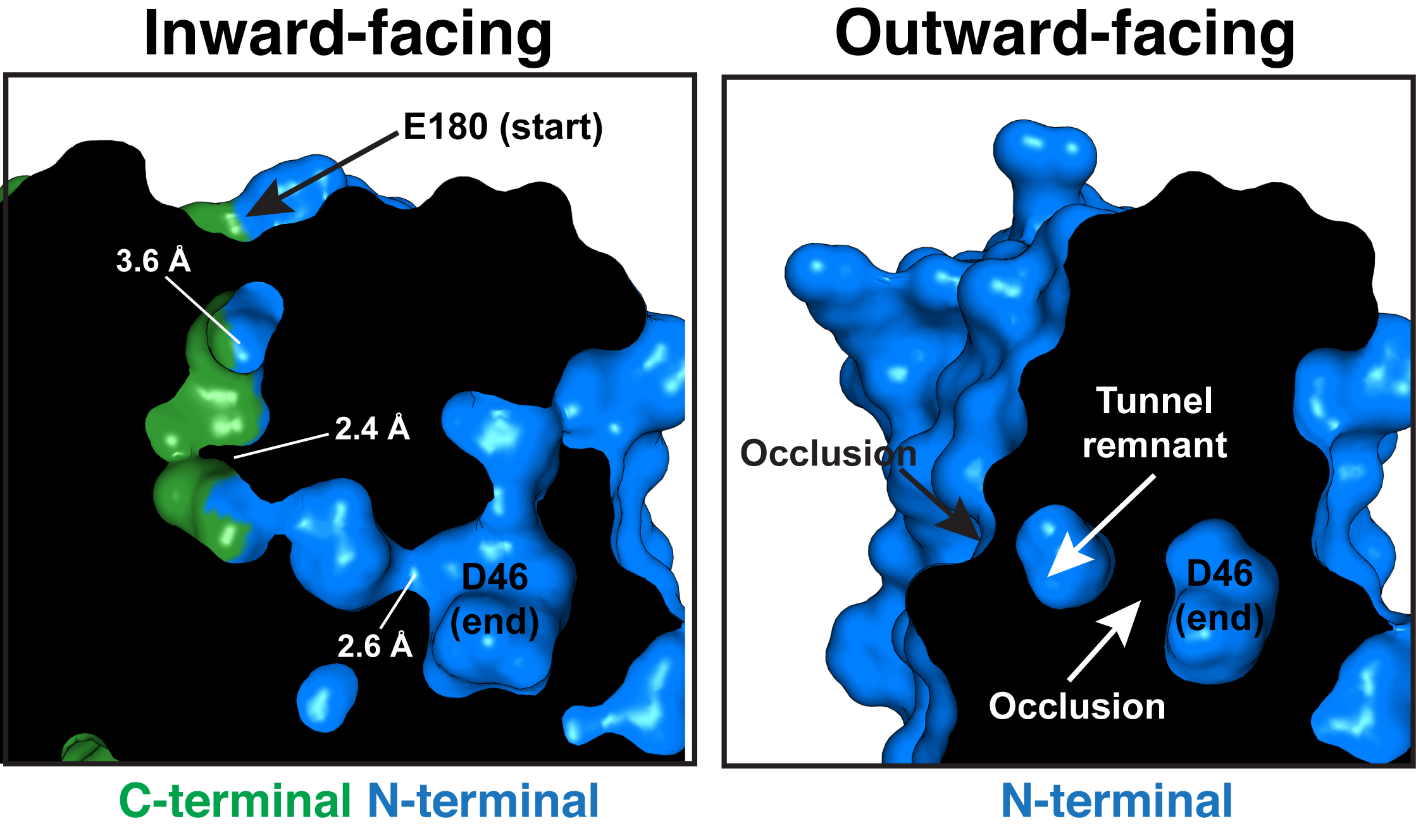

Supplement: S7 Fig — (Left panel) Cross section of inward-facing DgoT viewed from the periplasm shown in surface representation (N terminal: blue; C terminal: green). Arrow indicates the entrance to the putative H+ tunnel, ending at Asp46. (Right panel) Cross section of the N-terminal domain in the outward-facing conformation shows the remnant of the H+ tunnel with an occlusion. Asp46, Aspartate-46; DgoT, D-galactonate transporter. (TIF) [file pbio.3000260.s007.tif]

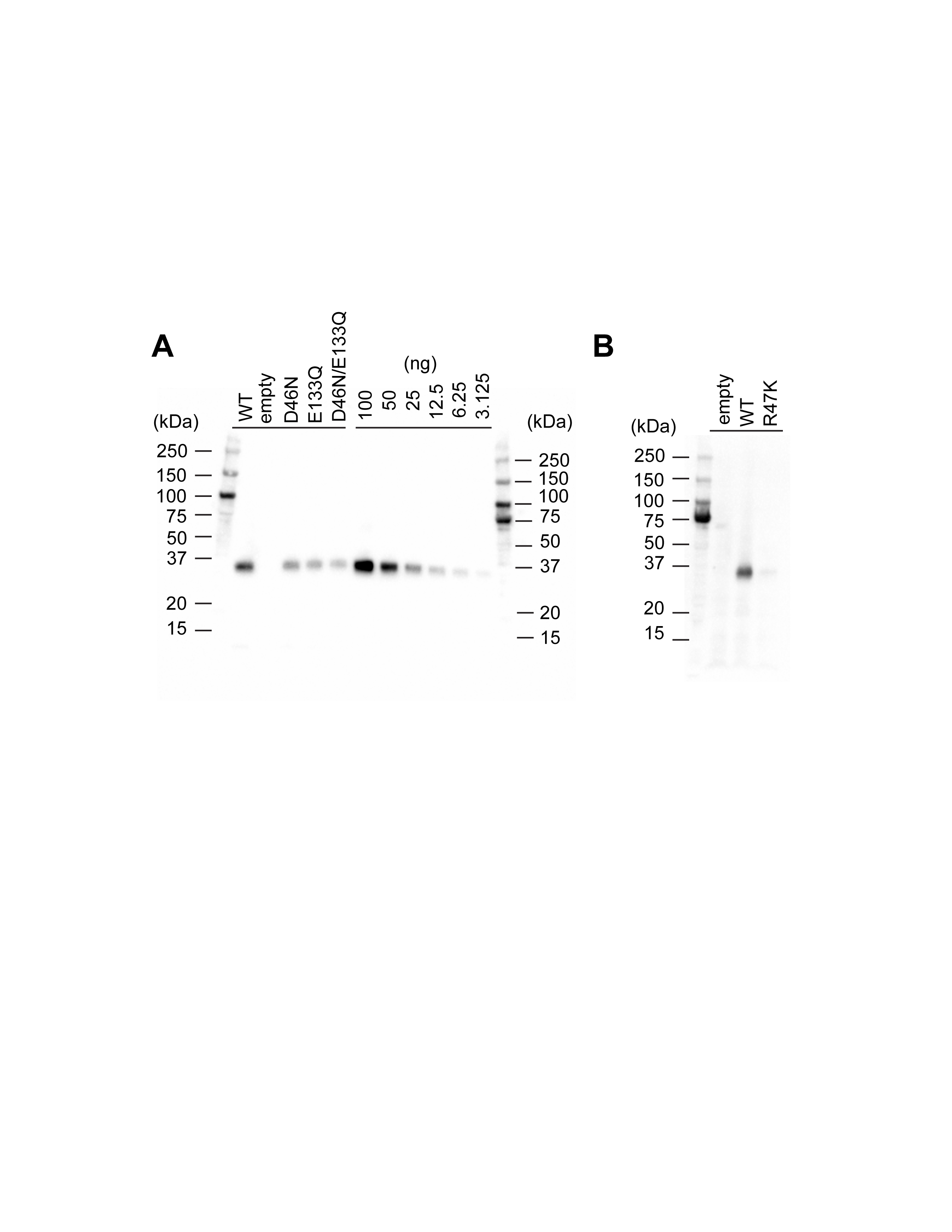

Supplement: S8 Fig — (A) Immunoblot of extracts from DgoT− E. coli transformed with WT E. coli DgoT, empty pQE60 vector, D46N, E133Q, or the double D46N/E133Q DgoT detected using a polyhistidine antibody and chemiluminescence. Standards of WT purified E. coli DgoT are shown to the right. (B) Immunoblot of extracts from DgoT− E.coli transformed with empty pQE60 vector, WT, and R47K DgoT. DgoT, D-galactonate transporter; WT, wild type. (TIF) [file pbio.3000260.s008.tif]
