# Supplementary material for: Structures suggest a mechanism for energy coupling by a family of organic anion transporters
Source: PLoS Biol. 2019 May 13;17(5):e3000260. doi: 10.1371/journal.pbio.3000260 (PMC6532931; doi:10.1371/journal.pbio.3000260)
Supplement: S1 Table — MR, molecular replacement. (DOCX) [file pbio.3000260.s009.docx]

|  | Inward-facing^a^ :wild type (6E9N) | Outward-facing^a^ :E133Q (6E9O) |
| --- | --- | --- |
| **Data collection** |  |  |
| Space group | P2_1_ | C2 |
| Cell dimensions |  |  |
| *a, b, c* (Å) | 76.7, 107.6, 103.2 | 217.1, 70.7, 107.2 |
| β, (°) | 108.3 | 101.8 |
| Resolution (Å) | 50.00 - 2.88 (3.00-2.88)^b^ | 106.23 - 3.49 (3.77-3.49)^b^ |
| *R*_meas_ | 0.11 (0.94)^c^ | 0.08 (5.15)^d^ |
| *<*I/σ(I)*>* | 10.1 (0.6)^e^ | 10.4 (0.2)^f^ |
| CC_1/2_  No. unique reflections  Completeness (%) | 99.9 (72.2)^g^  24356 (512)  95 to 3.8Å, 69.5 to 2.88Å (12.8); | 100 (15.9)^h^  20394 (4030)  99.8 (99.8) |
| Redundancy | 1.9 (1.3) | 2.0 (2.0) |
|  |  |  |
| **Refinement** |  |  |
| Resolution (Å) | 50- 2.9 * 3.8 * 3.8^i^ | 15 - 3.5 |
| No. reflections (work/free) | 21113/1254 | 18431/1843 |
| *R*_work_ / *R*_free_ | 24.9/29.7^j^ | 28.5/30.0^k^ |
| No. atoms |  |  |
| Protein | 6354 | 6138 |
| Ligand | 118 | 26 |
|  |  |  |
| *B*-factors |  |  |
| Protein | 57.0 | 203.5 |
| Ligand | 77.0 | 194.2 |
|  |  |  |
| R.m.s. deviations |  |  |
| Bond lengths (Å) | 0.003 | 0.004 |
| Bond angles (∘) | 0.57 | 0.99 |

^a^ Data for each set collected from a single crystal.

^b^ Highest resolution shell shown in parenthesis.

^c,d^ Redundancy of independent R factor calculated in HKL2000^c^ and XDS^d^, respectively.

^e^ If resolution cutoff is based on the criterion of *<* *I* /σ*I> >*1 the resolution is reduced to 3.5Å. However the quality of the electron density maps show details in places as in a 2.9Å density map.

^f^ If resolution cutoff is based on the criterion of *<* *I* /σ*I> >*1 the resolution is reduced to 4.0Å. The quality of details in better ordered regions of the map is as in a 3.5Å map.

^g,h^ The resolution for reporting data and refinement statistics is based on the statistically significant correlation coefficient value of CC_1/2_, i.e., the percentage of correlation between intensities from random half-datasets, as calculated in HKL2000^e^ and XDS^f^, respectively.

^i^ Elliptically truncated data used for final refinement.

^j^ 5.9% and ^k^10% of reflections were omitted from the refinement data for the calculation of R_free_, respectively.
